# Circulating immune and plasma biomarkers of time to HIV rebound in HIV controllers treated with vesatolimod

**DOI:** 10.3389/fimmu.2024.1405348

**Published:** 2024-06-24

**Authors:** Mohamed Abdel-Mohsen, Steven Deeks, Leila Giron, Kai Ying Hong, Aaron Goldman, Liao Zhang, Susie S. Y. Huang, Donovan Verrill, Susan Guo, Lisa Selzer, Christiaan R. de Vries, Elena Vendrame, Devi SenGupta, Jeffrey J. Wallin, Yanhui Cai

**Affiliations:** ^1^ Vaccine and Immunotherapy Center, The Wistar Institute, Philadelphia, PA, United States; ^2^ Department of Medicine, University of California, San Francisco, San Francisco, CA, United States; ^3^ Molecular and Cellular Oncogenesis Program, The Wistar Institute, Philadelphia, PA, United States; ^4^ Clinical Bioinformatics and Exploratory Analytics, Gilead Sciences, Inc., Foster City, CA, United States; ^5^ Statistical Programming, Gilead Sciences, Inc., Foster City, CA, United States; ^6^ Biostatistics, Gilead Sciences, Inc., Foster City, CA, United States; ^7^ Clinical Virology, Gilead Sciences, Inc., Foster City, CA, United States; ^8^ Clinical Development, Gilead Sciences, Inc., Foster City, CA, United States; ^9^ Biomarker Sciences and Diagnostics, Gilead Sciences, Inc., Foster City, CA, United States

**Keywords:** biomarkers, Analytic treatment interruption (ATI), vesatolimod, HIV-1, HIV controllers

## Abstract

**Background:**

Antiretroviral therapy (ART) for HIV-1 treatment has improved lifespan but requires lifelong adherence for people living with HIV (PLWH), highlighting the need for a cure. Evaluation of potential cure strategies requires analytic treatment interruption (ATI) with close monitoring of viral rebound. Predictive biomarkers for HIV-1 rebound and/or duration of control during ATI will facilitate these HIV cure trials while minimizing risks. Available evidence suggests that host immune, glycomic, lipid, and metabolic markers of inflammation may be associated with HIV-1 persistence in PLWH who are treated during chronic HIV-1 infection.

**Methods:**

We conducted *post-hoc* analysis of HIV controllers who could maintain low levels of plasma HIV-1 without ART in a phase 1b vesatolimod trial. Baseline and pre-ATI levels of immune, glycomic, lipidomic, and metabolomic markers were tested for association with ATI outcomes (time of HIV-1 rebound to 200 copies/mL and 1,000 copies/mL, duration of HIV-1 RNA ≤400 copies/mL and change in intact proviral HIV-1 DNA during ATI) using Spearman’s correlation and Cox proportional hazards model.

**Results:**

Higher levels of CD69+CD8+ T-cells were consistently associated with shorter time to HIV-1 rebound at baseline and pre-ATI. With few exceptions, baseline fucosylated, non-galactosylated, non-sialylated, bisecting IgG N-glycans were associated with shorter time to HIV rebound and duration of control as with previous studies. Baseline plasma MPA and HPA binding glycans and non-galactosylated/non-sialylated glycans were associated with longer time to HIV rebound, while baseline multiply-galactosylated glycans and sialylated glycans, GNA-binding glycans, NPA-binding glycans, WGA-binding glycans, and bisecting GlcNAc glycans were associated with shorter time to HIV rebound and duration of control. Fourteen bioactive lipids had significant baseline associations with longer time to rebound and duration of control, and larger intact proviral HIV-1 DNA changes; additionally, three baseline bioactive lipids were associated with shorter time to first rebound and duration of control.

**Conclusion:**

Consistent with studies in HIV non-controllers, proinflammatory glycans, lipids, and metabolites were generally associated with shorter duration of HIV-1 control. Notable differences were observed between HIV controllers vs. non-controllers in some specific markers. For the first time, exploratory biomarkers of ATI viral outcomes in HIV-controllers were investigated but require further validation.

## 1 Introduction

Antiretroviral therapy (ART) does not eliminate the latent HIV reservoir in people living with HIV (PLWH), consequently requiring lifelong adherence and making HIV cure a global public health objective (1, 2). The most robust and well accepted method to evaluate an HIV cure strategy is evaluating time of HIV rebound with an analytic treatment interruption (ATI), where ART is suspended and plasma HIV-1 RNA is monitored over time, often biweekly or weekly, to minimize risk to trial participants and threat of viral transmission to their partners (3). Identification of noninvasive biomarkers that can predict ATI viral outcomes (HIV rebound or control post-ART cessation) would enable more efficient and expedited clinical trial designs and mitigate the risk from ATI. Additionally, predictive biomarkers of ATI outcomes could help identify participants suitable for novel HIV cure strategies and facilitate the understanding of mechanisms of action (MOAs) that are associated with HIV clearance or control (3).

A small subset of PLWH who maintain low levels of viral replication (e.g., ≤400 copies/mL) after ART cessation (i.e. post-treatment controllers [PTC]), highlight the premise and constitute a model for HIV cure (4–6). However, no validated biomarkers currently exist that can distinguish PTC from non-PTC or predict HIV cure efficacy (7).

Viral and host factors associated with risk of HIV persistence and time to HIV rebound have been evaluated (4, 7, 8). Viral factors included size of the latent HIV reservoir, but quantity of replication-competent proviruses remains challenging to assess. Data from recent studies showed that changes from baseline in pre-ATI quantity of intact proviral HIV-1 DNA were found to be associated with time to viral rebound (9–11). Host factors related to immune and inflammatory responses were also found to be predictive of viral rebound and control (7). In a SPARTAC trial (EudraCT number, 2004-000446-20) analysis, CD4+ T-cell exhaustion markers prior to ART initiation were predictive of HIV rebound during ATI (12). Phenotypes and function of CD4+ and CD8+ T cells and innate immune cells such as natural killer (NK) or plasmacytoid dendritic cells (pDCs) may serve as other potential HIV rebound biomarkers (5, 13, 14). Interferon (IFN), IFN-stimulated genes (ISGs), inflammatory cytokines and chemokines, and other inflammation and immunoregulatory markers are actively being investigated as potential markers of HIV rebound or viral control (7, 15).

Recently, a novel category of non-invasive circulating biomarkers, including plasma glycoproteins, lipid and metabolites, were evaluated and found to be associated with HIV rebound during ATI in HIV non-controllers (16). Plasma glycoproteins and metabolites can enter the circulation from tissues through active secretion or translocation; therefore, their levels and the quantity and type of carbohydrates they exhibit can reflect the overall status of multiple tissues. Glycans on circulating glycoproteins, including antibodies, have functional significance for mediating immunologic functions, including antibody-dependent cell-mediated cellular cytotoxicity (ADCC) and pro- and anti-inflammatory responses (17). Circulating metabolites and bioactive lipids are also excellent candidate biomarkers for immune processes because they help modulate immune-cell function and inflammatory responses (18–21). *In vitro* studies also suggest that certain metabolites can modulate HIV latency reactivation and myeloid cell activation (4). Metabolic markers that were linked to ferroptosis were found associated with mitochondrial dysfunction and inflammation in CD4+ T cells (22). Some metabolites and lipids have been explored as biomarkers of HIV controllers and rapid progressors before and after seroconversion (8), and lipids are known to be involved in inflammatory complications of HIV infection (23, 24). Among those novel biomarkers, antibody glycosylation was also associated with time to viral rebound in HIV (4, 25); circulating tricarboxylic acid cycle metabolites, trimethylamine oxide, and tryptophan catabolism pathway metabolites were found to be associated with time to HIV rebound post ART cessation (4, 16). Circulating phospholipids and lysophospholipids were also associated with time to viral rebound and plasma viral load post ART cessation (16).

Strategies towards an HIV cure include approaches that can “CLEAR” HIV reservoir through direct targeting (e.g., broadly neutralizing antibodies) or “CONTROL” HIV-infected cells by protective immunity (e.g., vaccine). Vesatolimod, a highly selective and potent toll-like receptor 7 (TLR7) agonist, is under investigation to enhance immune responses against HIV infection in conjunction with “CLEAR” and/or “CONTROL” reagents. Vesatolimod has demonstrated efficacy in SHIV/macaque or SIV/macaque models by increasing time to viral rebound following ATI when combined with therapeutic vaccine and/or broadly neutralizing antibodies (26, 27). In HIV controllers, vesatolimod was found to decrease intact proviral DNA and modestly delay time to HIV rebound (10). The objective of this analysis was to assess the associations between baseline and pre-ATI immunologic and plasma glycomic, metabolomic, and lipidomic profiles with ATI viral outcomes and to determine the impact of vesatolimod vs. placebo on potential HIV rebound biomarkers identified in this study.

## 2 Methods

### 2.1 Study design and participants

Primary results of this placebo-controlled, phase 1b study (ClinicalTrials.gov identifier: NCT03060447) have been published (10). This study was undertaken in accordance with the Declaration of Helsinki and was approved by central or site-specific review boards or ethics committees. All participants provided written informed consent.

**Figure 1:**
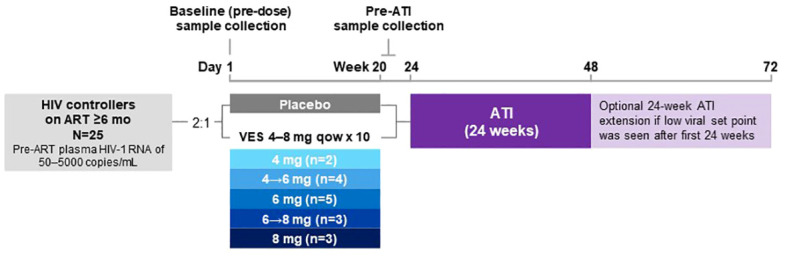
Study design. After screening, the participants were randomized 2:1 to receive vesatolimod or placebo, respectively, orally every 14 days for up to 10 doses. Based on data that emerged while the study was ongoing, the vesatolimod dose was increased from 4 mg to 6 mg, then to 8 mg. (10) There were a total of 7 participants who received vesatolimod with dose escalation for the consideration of improved efficacy while mitigating treatment-associated AEs. Four participants underwent vesatolimod dose escalation from 4 mg to 6 mg with two participants by dose 3, one by dose 5, and one by dose 7; three participants were dose escalated from 6 mg to 8 mg by dose 5, 6, and 8. Baseline samples were collected before the first vesatolimod dose on Day 1. Pre-ATI samples were collected 1 day after vesatolimod dose 10 for glycomic and lipid/metabolite data and to evaluate the vesatolimod effect, 7 days after for plasma cytokine data, and 13 days after for IFN-stimulated gene and immune-cell phenotyping data. AE, adverse event; ART, antiretroviral therapy; ATI, analytic treatment interruption; IFN, interferon; mo, months; qow, once every other week; VES, vesatolimod.

Twenty-five HIV viremic controllers who were on ART for ≥6 months with well-preserved CD4+ T cells were randomized 2:1 to vesatolimod doses of 4, 6, or 8 mg (n = 17) or placebo (n = 8, **Figure 1**). Two participants did not complete the study (1 participant decision; 1 incarcerated). Treatment arms were well balanced for participant age, gender, and CD4+ T-cell counts at baseline (**Table 1**). Vesatolimod provided a small but significant delay in time to viral rebound after ATI, and safety data supported that vesatolimod was well tolerated. See the primary report for further details (10).

**Table 1:** Summary of demographic, baseline key characteristics, and ATI viral outcomes of participants of vesatolimod vs. placebo arms.

	Vesatolimod (n = 17)	Placebo (n = 8)
Demographics and baseline key characteristics		
Age, years, median (range)	52 (31–66)	41 (27–53)
Male, n (%)	13 (77)	8 (100)
HIV-1 RNA (log_10_ copies/mL), median (IQR)	1.28 (1.28–1.28)	1.28 (1.28–1.28)
CD4+ T-cell count (cells/μL), median (IQR)	752 (550–915)	952 (652–1063)
Time to viral rebound after ATI		
Weeks to HIV-1 viral rebound to ≥200 copies/mL, median (IQR)	5.1 (4.1–6.3)*	4.1 (2.1–4.6)

*p = 0.024 vs. placebo.

### 2.2 Biomarker sampling and statistical analysis

Plasma, serum, and peripheral blood mononuclear cells were collected at baseline and multiple time points following treatment, and analyzed (10). Pre-ATI samples were collected following vesatolimod dose 10 (**Figure 1**).

The current analysis evaluated associations between candidate biomarker levels (at baseline and at pre-ATI) and ATI viral outcomes [time to plasma HIV-1 RNA first rebound to 200 or 1000 copies/mL, respectively; cumulative time of plasma viral load (pVL) ≤400 copies/mL during ATI (HIV control); and changes from baseline in the intact HIV-1 proviral DNA (changes from baseline in the HIV reservoir)]. Associations were assessed using Spearman’s correlation analysis and Cox proportional hazards model using a median split to test associations with HIV control measures for biomarker levels above and below the study median. Wilcoxon signed-rank test and rank sum test were used to compare between time points and populations. Analyses were not corrected for multiple comparisons due to small sample size, and nominal p-value was used to evaluate significance. Data from the vesatolimod and placebo groups were pooled (all were HIV controllers), but the 2:1 randomization skewed the results toward the vesatolimod group, which had a modestly longer time to rebound during ATI in the main study. Enrichment-ratio for metabolic pathways was computed by observed hits/expected hits; nominal p values are presented.

### 2.3 Biomarker analytes

Immune biomarkers evaluated included markers of immune cell activation and vesatolimod pharmacodynamic cytokines/chemokines (10). Evaluation of immune cell activation included assessment of the following cellular subsets: NK cells (CD16+ and CD69+), monocytes (CD14+CD16+), CD8+ T cells (Ki67+, CD69+, CD38+, and HLA-DR+CD38+), and CD4+ T cells (Ki67+, CD69+, and HLA-DR+CD38+); geometric mean fluorescent intensity of CD40 and CD54 on plasmacytoid dendritic cells; plasma cytokines: interleukin (IL)-1 receptor antagonist, IFN-γ–induced protein 10, IFN-inducible T-cell alpha chemoattractant (ITAC), and IFN-α.

The sets of glycomic, lipidomic, and metabolomic biomarkers assessed were based on those evaluated in prior studies of biomarkers of HIV viral dynamics during ATI (4, 16, 28).

### 2.4 Viral load and biomarker assessment assays

pVL was measured by quantitative PCR (Roche TaqMan 2.0 assay; Covance, Indianapolis, IN). Intact proviral HIV-1 DNA was measured by the intact HIV proviral DNA assay (AccelevirDx, Baltimore, MD) (29). Immune-cell phenotyping was performed using flow cytometry (UCSF, San Francisco, CA). Plasma and isolated immunoglobulin G (IgG) glycomic profiles were analyzed using lectin array and capillary electrophoresis (Wistar Institute, Philadelphia, PA) as in a previous report (4). Serum concentrations of IFN γ-induced protein 10 kDa (IP10), IL-1RA, and IFN-α were quantified using high-sensitivity Ciraplex assays (Aushon Biosystems, Billerica, MA). Plasma metabolites and lipids were measured by HPLC-MS/MS (Wistar Institute, Philadelphia, PA). Pathway enrichment analysis was referred to a library of 84 metabolite sets based on Kyoto Encyclopedia of Genes and Genomes human metabolic pathways (Oct. 2019); https://www.metaboanalyst.ca.

## 3 Results

### 3.1 Baseline biomarkers

#### 3.1.1 Immune biomarkers

**Figure 2:**
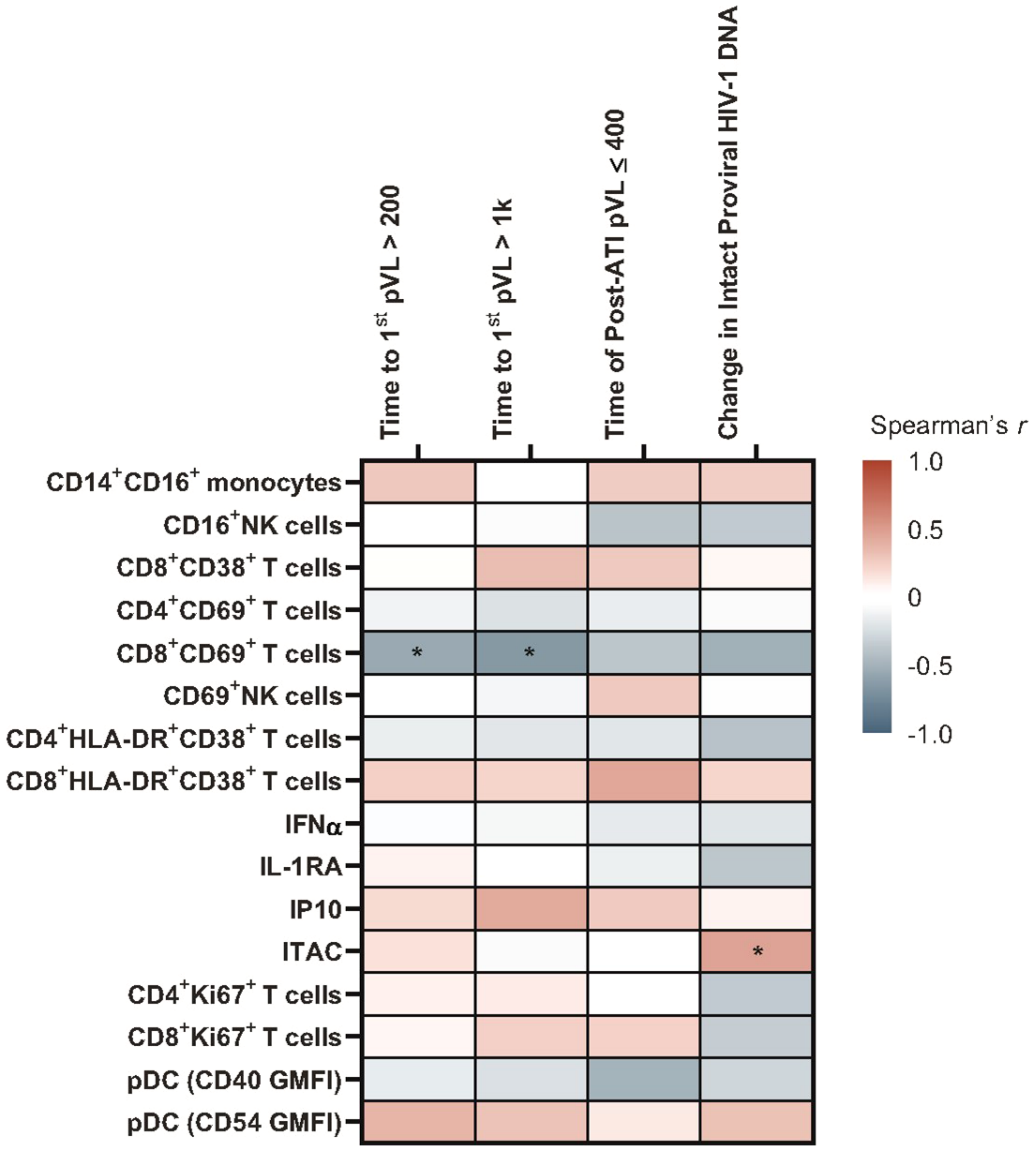
Heatmap of associations between baseline immune biomarkers and HIV control post ART-cessation. Data used for the analysis include the pooled vesatolimod and placebo groups and are limited to the analytes that pass quality control; for flow-based assays, cutoff is 70% viability. *p <0.05. GMFI, geometric mean fluorescent intensity.

ITAC positively correlated with change in intact HIV-1 proviral DNA from baseline (r = 0.46, p = 0.048), and the proportion of CD69+CD8+ T cells was negatively associated with time to first HIV rebound to 200 copies/mL (r = –0.549, p = 0.05) and 1000 copies/mL (r = –0.642, p = 0.02) using Spearman’s correlation. Neither biomarker achieved statistical significance in the Cox proportional hazards model (**Figure 2**; **Supplementary Tables 1, 2**).

#### 3.1.2 Glycomic biomarkers

**Figure 3:**
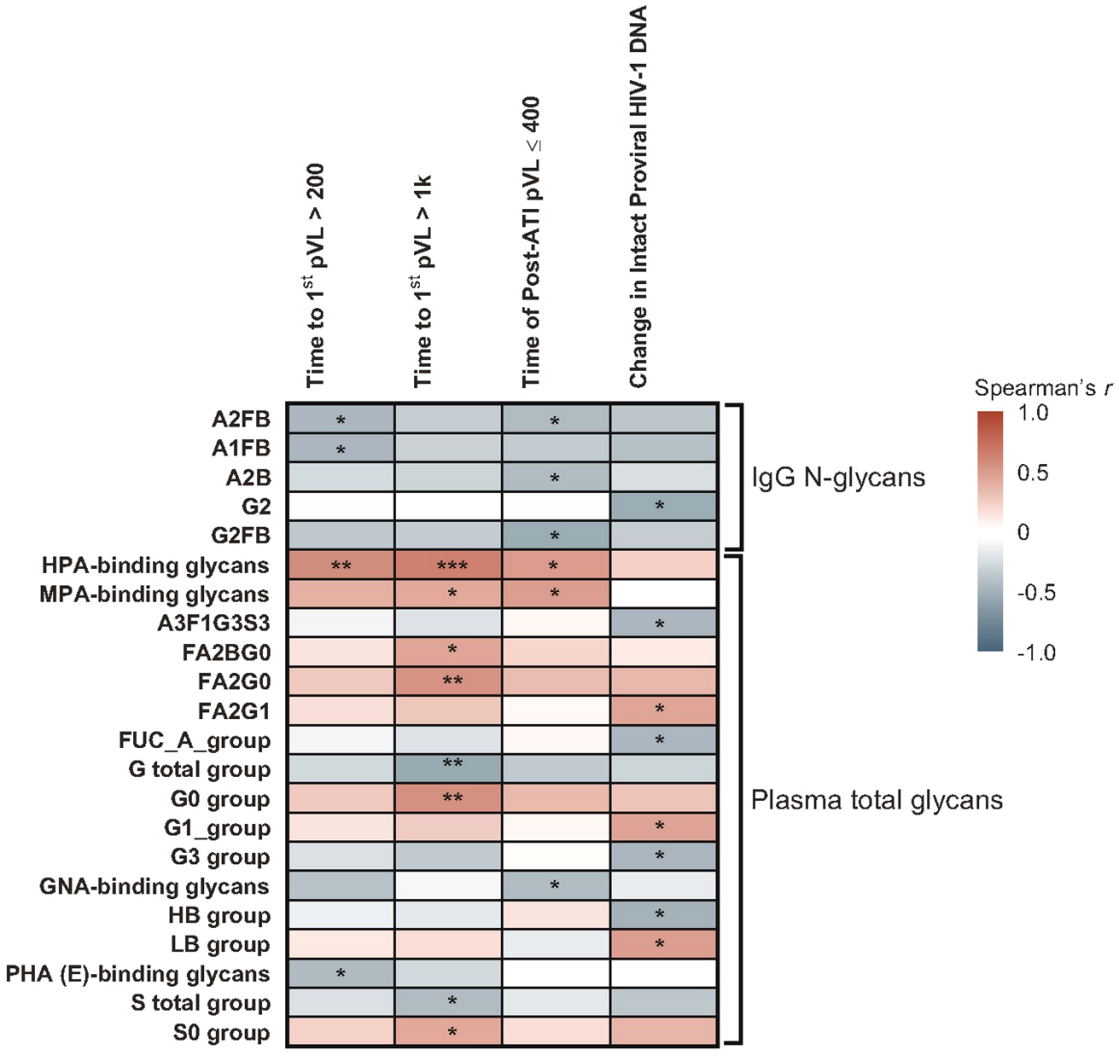
Heatmap of associations between baseline glycomic biomarkers and HIV control post ART-cessation. Data include only participants with nominal p ≤0.05 for ≥1 plasma HIV-1 RNA copies/mL level in the pooled vesatolimod and placebo groups and are limited to the analytes that pass quality control. *p <0.05; **p <0.01; ***p <0.001.

**Figure 4:**
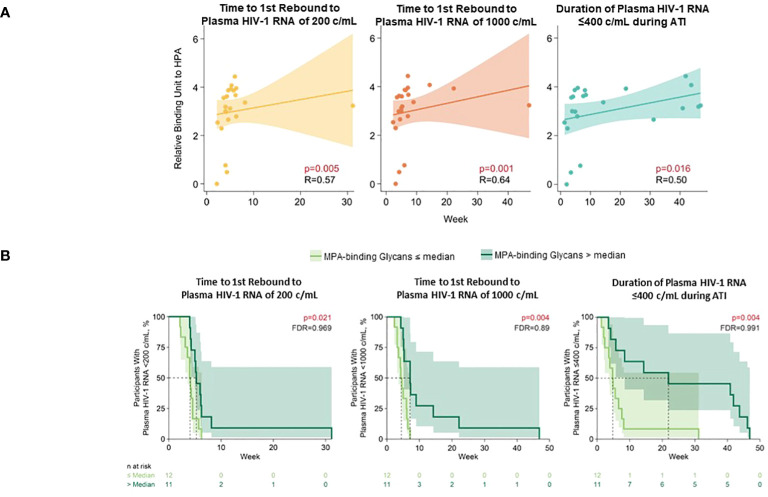
Higher baseline HPA- and MPA-binding glycans associated with longer time to HIV rebound and duration of HIV control during ATI. **(A)** HPA-binding glycans analyzed using Spearman’s correlation. **(B)** MPA-binding glycans analyzed using Cox proportional hazards model. Nominal p-value and correlation coefficient (r) are reported. Data used for the analyses include the pooled vesatolimod and placebo arms; analyses are limited to the analytes that pass quality control.

Several glycomic biomarkers were positively associated with HIV control during ATI using Spearman’s correlation analysis (**Figure 3**; **Supplementary Table 3**). Plasma *Helix pomatia* agglutinin (HPA)-binding glycans positively correlated with time to first HIV rebound to 200 (p = 0.005) and 1000 copies/mL (p = 0.001) and cumulative time of pVL ≤400 copies/mL during ATI (p = 0.016). *Maclura pomifera* agglutinin (MPA)-binding glycans positively correlated with time to first HIV rebound to 1000 copies/mL (p = 0.041) and cumulative time of pVL ≤400 copies/mL during ATI (p = 0.019). FA2BG0 (p = 0.037), FA2G0 (p = 0.009), G0 group (p = 0.008), and S0 group (p = 0.045) glycans were also positively correlated with time to first HIV rebound to 1000 copies/mL. FA2G1 (p = 0.039), G1 (p = 0.038), and low-branched (LB; p = 0.025) groups were positively associated with change in intact HIV-1 proviral DNA. Higher baseline HPA-binding glycans were significantly associated with longer time to first HIV rebound to 200 copies/mL (p = 0.005) and 1000 copies/mL (p = 0.001), and cumulative time of pVL ≤400 copies/mL during ATI (p = 0.016; **Figure 4A**). MPA-binding glycans greater than the median was a positive correlate of time to first rebound to 200 (p = 0.021) and 1000 (p = 0.004) copies/mL, and cumulative time of pVL ≤400 copies/mL (p = 0.004) with Cox proportional hazard model (**Figure 4B**; **Supplementary Table 4**).

On the other hand, several glycomic biomarkers that negatively associated with a measure of HIV control included IgG glycans associated with time to first rebound to 200 copies/mL (A2FB [p = 0.028] and A1FB [p = 0.029]), cumulative time of pVL ≤400 copies/mL (A2FB [p = 0.043], A2B [p = 0.048], G2FB [p = 0.011]), or change in intact HIV-1 proviral DNA (G2 [p = 0.017]). Plasma glycans included those correlated with time to first rebound to 200 copies/mL (*Phaseolus vulgaris* phytohemagglutinin [PHA{E}]-binding [p = 0.035]), time to first HIV rebound to 1000 copies/mL (total galactosylated [G] group [p = 0.008], total sialylated [S] group [p = 0.045]), cumulative time of pVL ≤400 copies/mL (GNA-binding [p = 0.040]), or change in intact HIV-1 proviral DNA (A3F1G3S3 [p = 0.032], total antennary fucosylated (Fuc-A) [p = 0.032], tri-galactosylated (G3) [p = 0.031], high-branched (HB) group [p = 0.025]) (**Supplementary Table 3**).

In the Cox proportional hazards model, negative associations with duration of HIV control or time to HIV rebound were found for IgG glycans A2B (p = 0.013) and G1FB (p = 0.050); and plasma glycans A3G3S3 (time to first HIV rebound to 200 copies/mL: p = 0.037 and 1000 copies/mL: p = 0.028), G2 (time to first HIV rebound to 1000 copies/mL: p = 0.036 and cumulative time of pVL ≤400 copies/mL: p = 0.029), *Griffania simplicifolia* lectin (GSL) II glycans (p = 0.029), *Narcissus pseudonarcissus* agglutinin (NPA)-binding (p = 0.050), and wheat-germ agglutinin (WGA)-binding (p = 0.011) groups (**Supplementary Table 4**).

#### 3.1.3 Lipid/metabolite biomarkers

**Figure 5:**
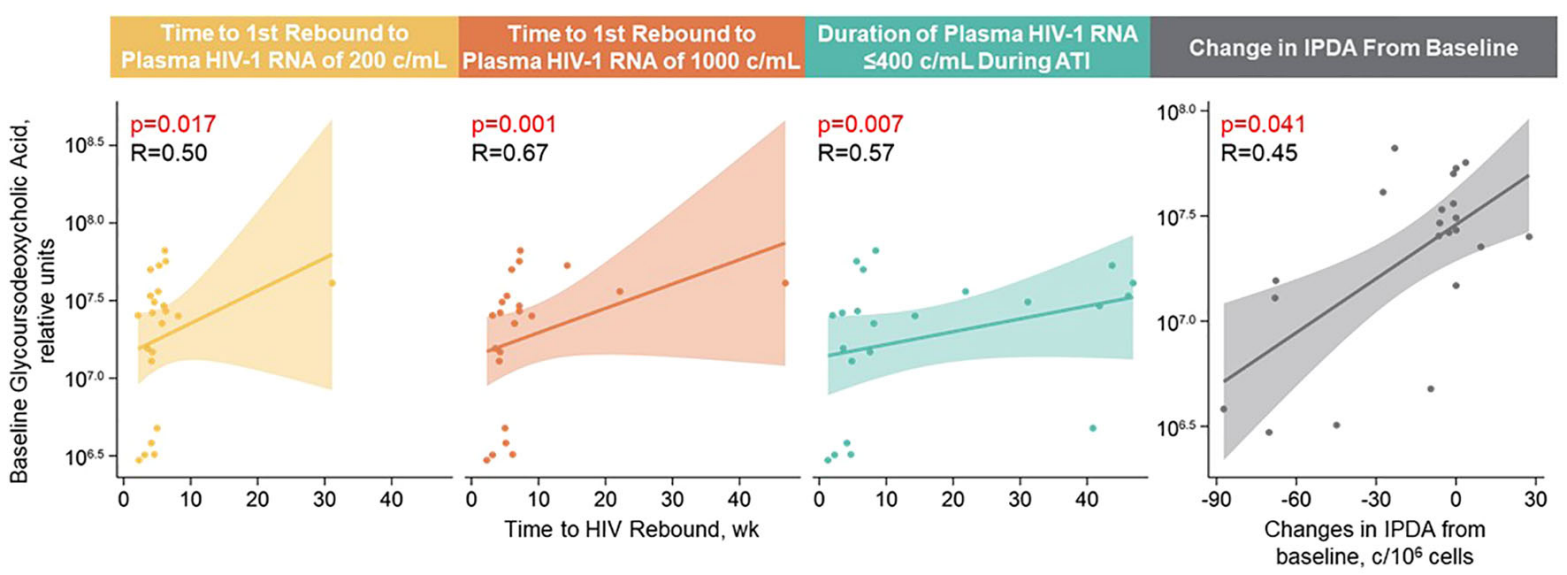
Higher baseline glycoursodeoxycholic acid levels correlated with longer time to HIV rebound, longer control during ATI, and larger intact HIV-1 proviral DNA changes. Spearman’s correlation was used; data used for the analyses include the pooled vesatolimod and placebo arms; analyses are limited to the analytes that pass quality control.

**Figure 6:**
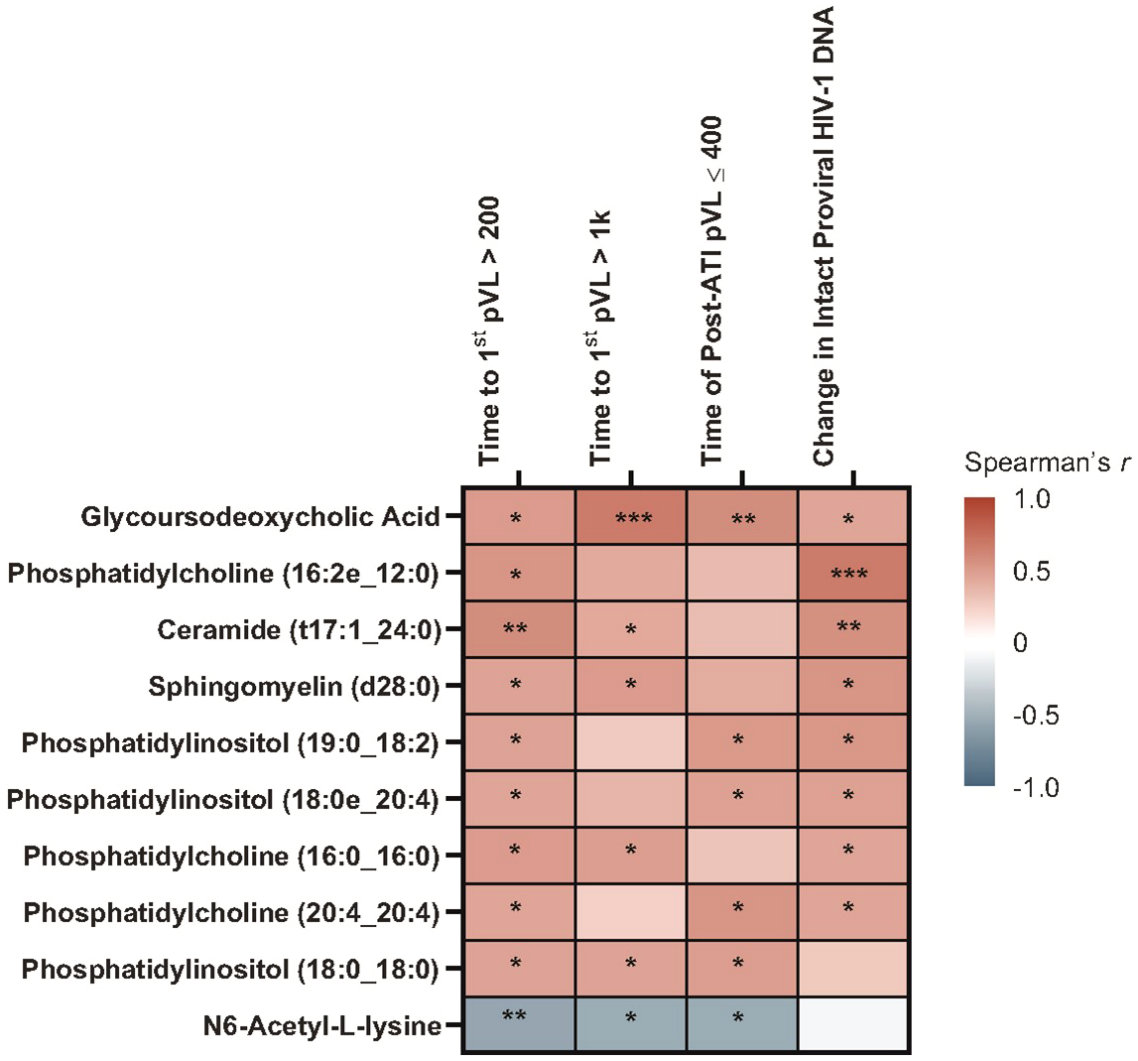
Heatmap of Spearman’s correlations between baseline lipid/metabolite biomarkers and time to HIV rebound and duration of HIV control during ATI. Data include participants with nominal p ≤0.05 for ≥3 viral outcomes in the pooled vesatolimod and placebo groups and are limited to the analytes that pass quality control. *p <0.05, **p <0.01, ***p <0.001.

With Spearman’s correlation analysis, higher baseline glycoursodeoxycholic acid levels correlated with longer time to first HIV rebound to 200 copies/mL (p = 0.017) and 1000 copies/mL (p = 0.001), longer cumulative time of pVL ≤400 copies/mL during ATI (p = 0.007), and larger intact HIV-1 proviral DNA changes (p = 0.041; **Figure 5**; **Supplementary Table 5**). Additional lipid and metabolite biomarkers significantly positively associated with time to first HIV rebound to 200 copies/mL and at least one other measure of control included phosphatidylcholines (16:2e_12:0) [p = 0.011, 0.050, not significant {NS}, 0.001]; (16:0_16:0) [p = 0.017, 0.022, NS, 0.039]; (20:4_20:4) [p = 0.038, NS, 0.011, 0.041], ceramide (t17:1_24:0) [p = 0.006, 0.046, NS, 0.010], sphingomyelin (d28:0) [p = 0.030, 0.018, NS, 0.014], and phosphatidylinositol (19:0_18:2) [p = 0.031, NS, 0.016, 0.015]; (18:0e_20:4) [p = 0.038, NS, 0.028, 0.030]; (18:0_18:0) [p = 0.033, 0.030, 0.022, NS]. All of these but phosphatidylinositol (18:0_18:0) were also significantly associated with change in intact HIV-1 proviral DNA. The only biomarker that was negatively associated with cumulative time of pVL ≤400 copies/mL was N6-acetyl-L-lysine (p = 0.006, 0.012, 0.013, NS; **Figure 6**; **Supplementary Table 5**).

**Figure 7:**
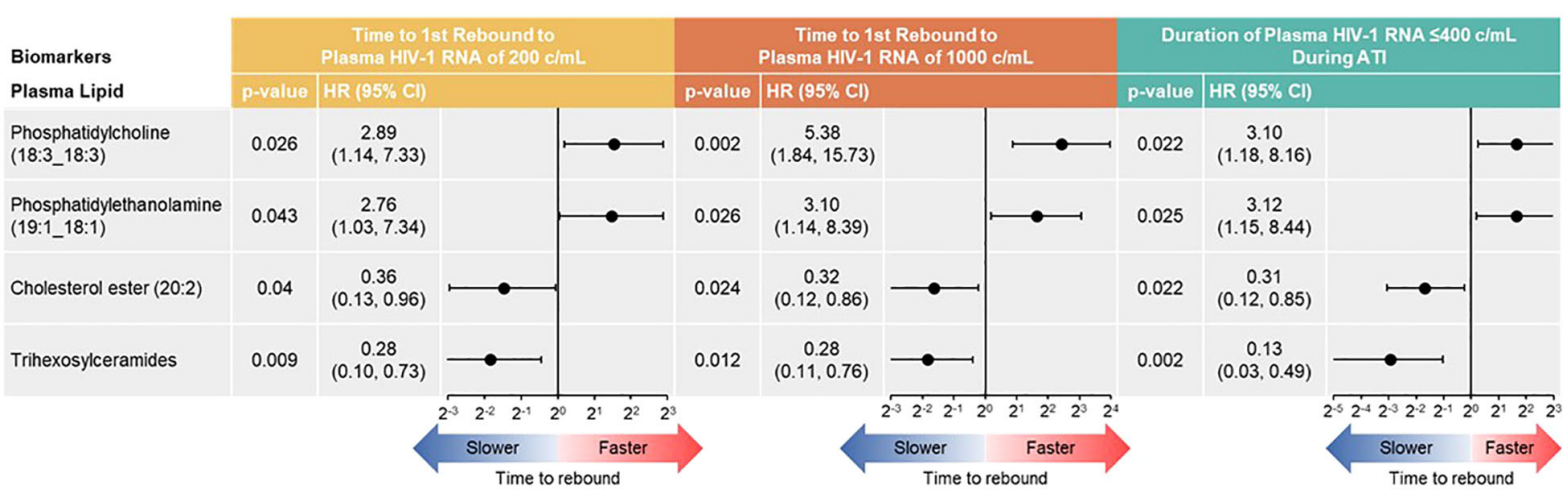
Baseline lipid/metabolite biomarkers correlated with time to HIV rebound and duration of HIV control during ATI. Correlation assessed with Cox proportional hazards model. Data used for the analyses include participants with nominal p ≤0.05 for ≥3 viral outcomes in the pooled vesatolimod and placebo arms; analyses are limited to the analytes that pass quality control.

With the Cox proportional hazards model (**Figure 7**), cholesterol ester (20:2) and trihexosylceramide were significantly associated with longer time to first plasma HIV-1 RNA rebound to 200 copies/mL (p = 0.040 and 0.009, respectively) and to 1000 copies/mL (p = 0.024 and 0.012, respectively); and longer cumulative time with pVL ≤400 copies/mL during ATI, (p = 0.022 and 0.002, respectively). Phosphatidylcholine (18:3_18:3) and phosphatidylethanolamine (19:1_18:1) were associated with shorter time to rebound to plasma HIV-1 RNA of 200 copies/mL (p = 0.026 and 0.043, respectively) and to 1000 copies/mL (p = 0.002 and 0.026, respectively), and shorter cumulative time of pVL ≤400 copies/mL during ATI (p = 0.022 and 0.025, respectively).

Protein synthesis–related pathways were also associated with HIV rebound. Upregulation of pantothenate and coenzyme A biosynthesis and beta-alanine metabolism correlated with time to first-time HIV-1 rebound to 200 copies/mL but not 1000 copies/mL (**Supplementary Figure 1**).

### 3.2 Pre-ATI biomarkers

#### 3.2.1 Immune biomarkers

Vesatolimod activates innate immune responses through TLR7-signaling, leading to IFN-stimulated gene expression, cytokine release, and activation of T cells and NK cells (30), which may affect biomarker levels transiently with return to a level close to baseline after a period of time (~13 days) (10). Immune biomarkers analyzed in this study included baseline and pre-ATI time points (7 days after vesatolimod dose 10 for plasma cytokines and 13 days after for immune-cell phenotyping data).

**Figure 8:**
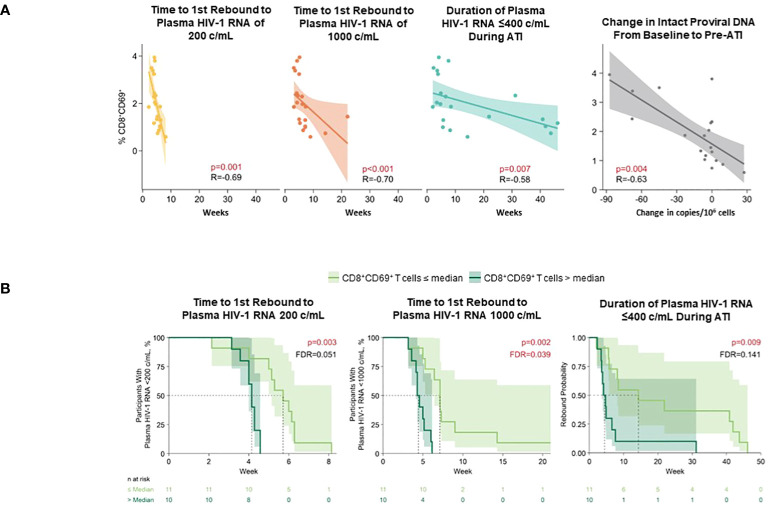
Higher level of pre-ATI CD69^+^CD8^+^ T cells correlated with shorter time to HIV rebound, shorter duration of HIV control during ATI, and smaller changes in intact HIV-1 proviral DNA. **(A)** Spearman’s correlation. Nominal p-value and R are reported. **(B)** Cox proportional hazards model. Data used for the analyses include participants with nominal p ≤0.05 for ≥3 viral outcomes in the pooled vesatolimod and placebo arms; analyses are limited to the analytes that pass quality control. FDR, false discovery rate.

A higher level of pre-ATI CD69+CD8+ T cells strongly correlated with shorter time to first HIV-1 rebound to 200 (p = 0.001) and 1000 copies/mL (p <0.01), shorter cumulative time of pVL ≤400 copies/mL during ATI (p ≤0.007), and smaller changes in intact HIV-1 proviral DNA (p = 0.004) using Spearman’s correlation; in the Cox proportional hazards model, corresponding p-values for time to HIV rebound to 200 and 1000 copies/mL were p = 0.003 and p = 0.002, respectively, and p = 0.009 for cumulative time of pVL ≤400 copies/mL (**Figures 8A, B**). CD54+pDC positively associated with time to first HIV rebound to 200 copies/mL (p = 0.05) but no other measure of HIV control using Spearman’s correlation (**Supplementary Table 6**). Additionally, negative association of CD69+CD8+ T cells with all measures of HIV control was also detected in the Cox proportional hazards model (**Supplementary Table 7**).

#### 3.2.2 Glycomic biomarkers

**Figure 9:**
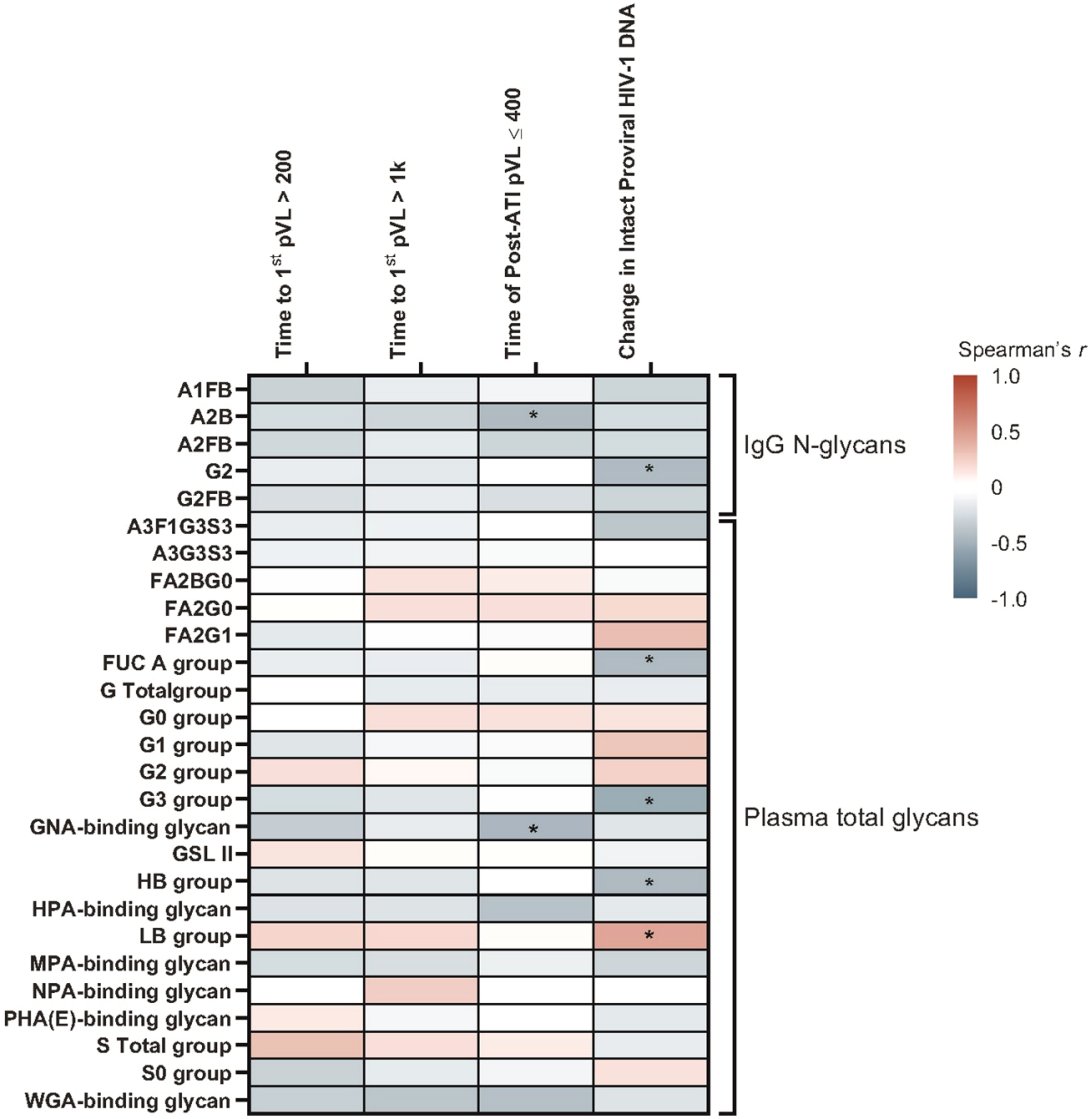
A subset of glycomic biomarkers remained associated with HIV rebound in the pre-ATI analysis. Data used for the analysis include the pooled vesatolimod and placebo arms and are limited to the analytes that pass quality control. Nominal p-values are presented. *p <0.05.

At the pre-ATI time point, IgG N-glycan A2B (p = 0.036) and plasma GNA binding glycan (p = 0.025) were negatively associated with cumulative time of pVL ≤400 copies/mL during ATI using Spearman’s correlation (**Figure 9**; **Supplementary Table 8**). LB group had a significant positive association with change in intact HIV-1 proviral DNA (p = 0.039). Several other glycomic biomarkers were negatively associated with change in intact HIV-1 proviral DNA: IgG N-glycan G2 (p = 0.045), and plasma glycan groups antenna-fucosylated (FUC_A; p = 0.046), G3 (p = 0.013), and HB (p = 0.039) groups.

In the Cox proportional hazards model, pre-ATI IgG N-glycan A2B and plasma GNA-binding glycan were negatively associated with cumulative time of pVL ≤400 copies/mL during ATI, with an A2B hazard ratio (HR) = 2.5 (95% confidence interval [CI]: 1.0, 6.4; p = 0.05) and GNA-binding glycans HR = 2.5 (95% CI: 1.1, 6.2; p = 0.038) (**Supplementary Table 9**).

#### 3.2.3 Lipid/metabolite biomarkers

**Figure 10:**
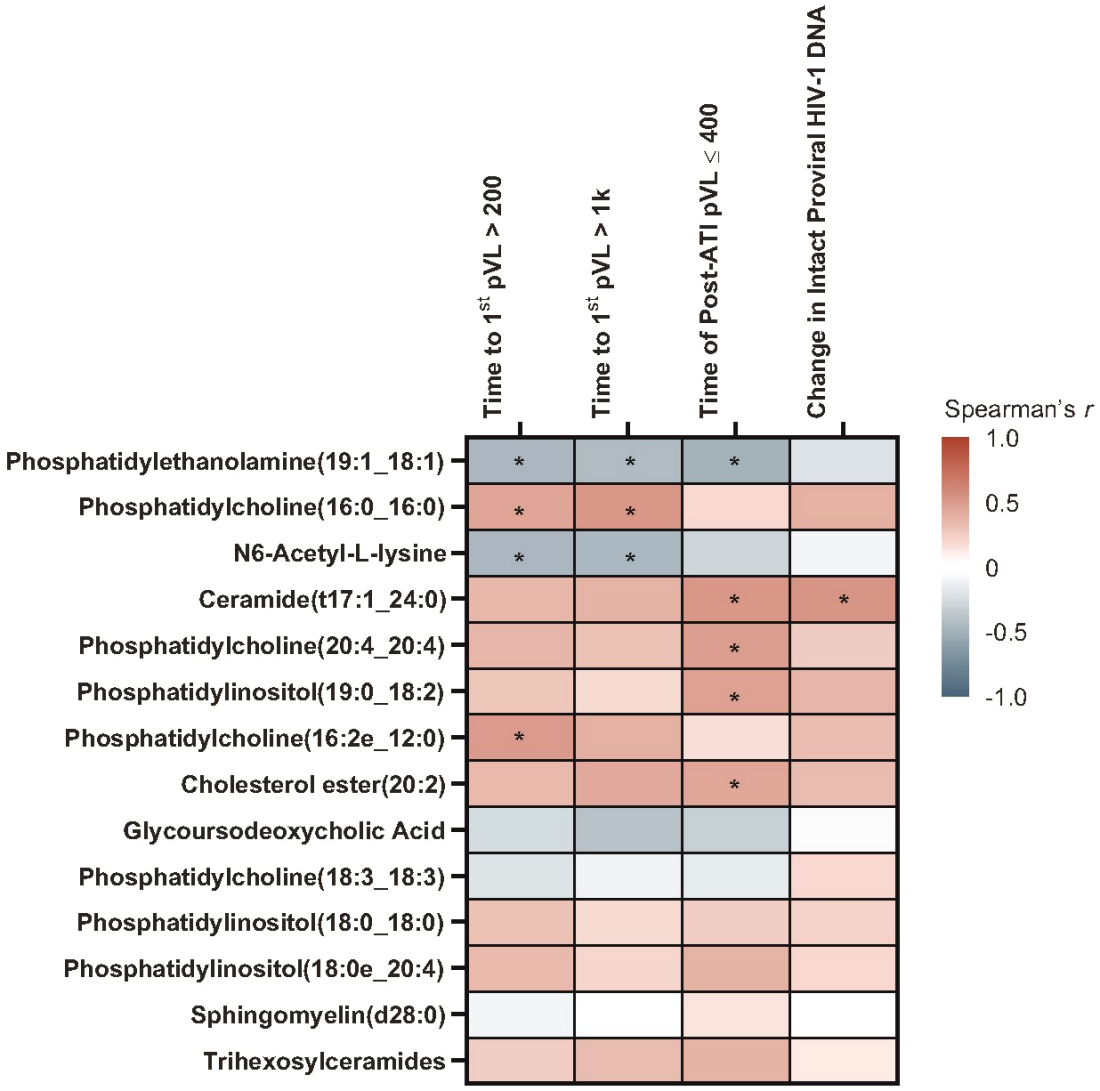
A subset of pre-ATI lipid/metabolite biomarkers remained associated with HIV rebound during ATI. Data used for the analysis include the pooled vesatolimod and placebo arms and are limited to the analytes that pass quality control. Nominal p-values are presented. *p <0.05.

Pre-ATI lipid/metabolite biomarkers with positive associations using Spearman’s correlation (**Figure 10**; **Supplementary Table 10**) with time to first HIV rebound to 200 copies/mL were phosphatidylcholines (16:0_16:0) [p = 0.034] and (16:2e_12:0) [p = 0.017]. Phosphatidylcholine (16:0_16:0) was also positively associated with time to first HIV rebound to 1000 copies/mL (p = 0.013). Ceramide (t17:1_24:0) [p = 0.015], phosphatidylcholine (20:4_20:4) [p = 0.022], phosphatidylinositol (19:0_18:2) [p = 0.028], and cholesterol ester (20:2) [p = 0.042] were positively associated with cumulative time of pVL ≤400 copies/mL. Ceramide (t17:1_24:0) was also positively associated with changes in intact HIV-1 proviral DNA from baseline (p = 0.013). N6-acetyl-L-lysine and phosphatidylethanolamine (19:1_18:1) had negative associations with time to first HIV rebound to 200 copies/mL (p = 0.028 and 0.026, respectively) and 1000 copies/mL (p = 0.029 and 0.045, respectively); phosphatidylethanolamine (19:1_18:1) was also negatively associated with cumulative time of pVL ≤400 copies/mL during ATI (p = 0.021).

In the Cox proportional hazards model, positive associations were found with trihexosylceramides, which were significant for all three measures of HIV control (time to first HIV rebound to 200 copies/mL, p = 0.021; time to first HIV rebound to 1000 copies/mL, p = 0.018; cumulative time of pVL ≤400 copies/mL, p = 0.017). Other positive associations were phosphatidylcholine (16:0_16:0), with time to first HIV rebound to 200 copies/mL (p = 0.033) and 1000 copies/mL (p = 0.020); phosphatidylcholine (16:2e_12:0), with time to first rebound to 200 copies/mL (p = 0.021); and phosphatidylcholine (20:4_20:4) [p = 0.014], ceramide (t17:1_24:0) [p = 0.048], phosphatidylinositol (19:0_18:2) [p = 0.008], and cholesterol ester (20:2) [p = 0.017] with cumulative time of pVL ≤400 copies/mL (**Supplementary Table 11**). Interestingly, glycoursodeoxycholic acid, which was associated with all measures of HIV control and intact HIV-1 proviral DNA in the baseline biomarker set, was not significantly associated with any measure in either analysis of the pre-ATI biomarker set.

### 3.3 Biomarkers with vesatolimod treatment

The impact of vesatolimod treatment vs. placebo on biomarker levels was explored in baseline and pre-ATI assessments. Among immune biomarkers, levels of CD14+CD16+ monocytes were significantly higher in the vesatolimod treatment vs. placebo groups by Wilcoxon rank sum test at both baseline (1.68 log_2_ fold-change; p = 0.008) and pre-ATI time points (1.55 log_2_ fold-change; p = 0.016) (**Supplementary Table 12**). No differences were observed at baseline or pre-ATI between vesatolimod and placebo groups in any other biomarkers (**Supplementary Tables 13, 14**).

**Figure 11:**
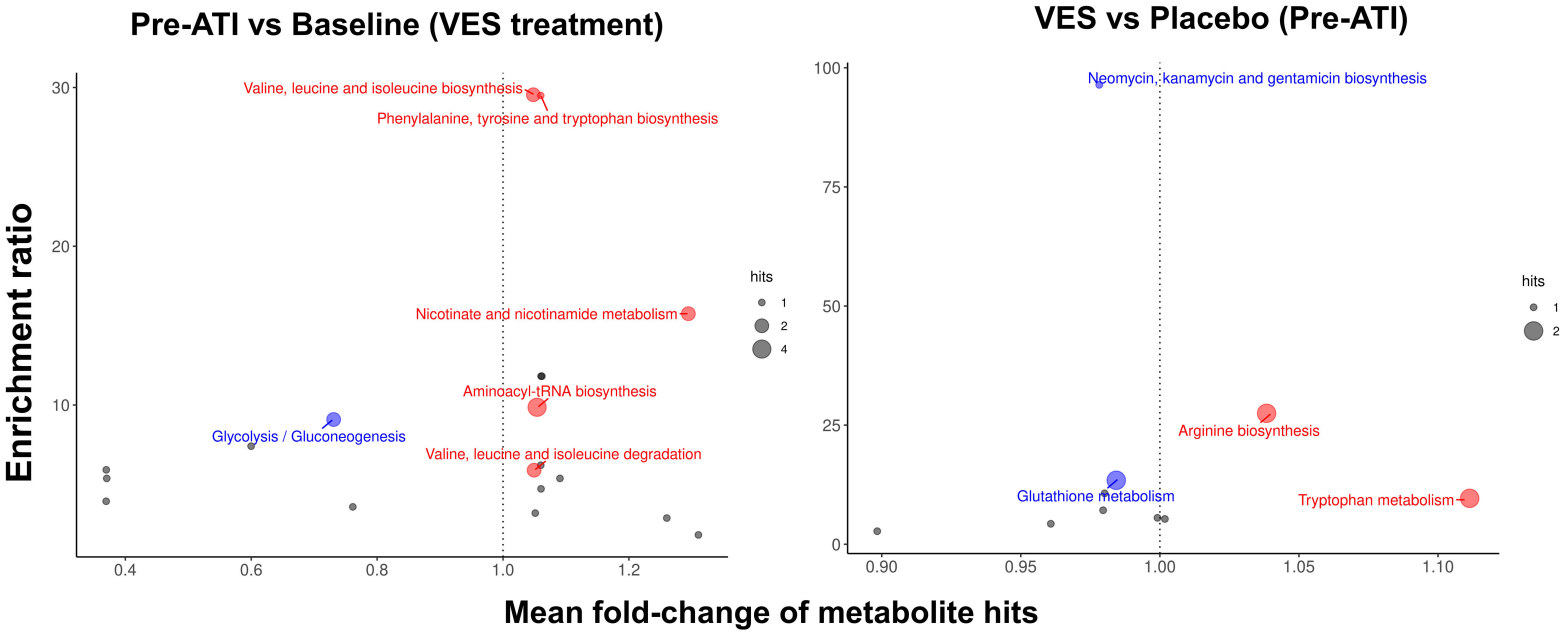
Pro-inflammatory tryptophan metabolism and protein synthesis–related pathways are enriched after vesatolimod treatment. Dot size represents the number of hits. Dot color represents upregulation (red, mean fold-change >1), downregulation (blue, mean fold-change <1), and nonsignificant (gray). Fold change is shown on the *x*-axis. Nominal p-values were used in the analysis. VES, vesatolimod.

Pro-inflammatory tryptophan metabolism and protein synthesis–related pathways were enriched after vesatolimod treatment. Enrichment ratios (pre-ATI vs. baseline) were largest with valine, leucine, isoleucine biosynthesis; phenylalanine, tyrosine, tryptophan biosynthesis; and nicotinate, nicotinamide metabolism (**Figure 11**; **Supplementary Figure 2**). Aminoacyl-transfer RNA (tRNA) biosynthesis related to protein synthesis had the largest statistically significant association.

Downregulation of glutathione metabolism and neomycin, kanamycin, and gentamicin biosynthesis showed the greatest enrichment with vesatolimod treatment vs. placebo pre-ATI, followed by increased arginine biosynthesis and tryptophan metabolism (**Figure 11**; **Supplementary Figure 2**). Tryptophan metabolism had the highest statistically significant enrichment. Vesatolimod treatment mediated significant changes in more lipid subclasses than placebo did (**Supplementary Figure 3**). Diacylglycerols, lysophosphatidylcholine, lysophosphatidylinositol, phosphatidic acid, and phosphatidylserine changes were all unique to vesatolimod; the significant change in acyl carnitine was unique to placebo.

## 4 Discussion

Although research on biomarkers of ATI HIV rebound or control is in its infancy, increasing work has been done and focused almost exclusively on the pre-ATI timepoint in PLWH treated during chronic infection (4, 12, 16, 25, 28, 31). Findings from these studies suggest that, in addition to markers of T-cell exhaustion, proinflammatory glycan structures, phospholipid species, and metabolites are associated with more rapid HIV in PLWH (4, 12, 16, 25, 28, 31). HIV controllers, a distinct category of PLWH, were often studied to understand potential mechanisms of HIV control, but none of the work has ever evaluated HIV rebound biomarkers in this unique population (6). In this study, we analyzed ATI viral outcome biomarkers from baseline and pre-ATI time points, and found some unique and some shared glycomic, lipid and metabolite biomarkers associated with ATI HIV outcomes compared with those reported in HIV non-controllers.

Among all the immune biomarkers evaluated in this study, only baseline CD69+CD8+ T-cell and ITAC levels reached significance. Interestingly, CD69+CD8+ T cells retained associations with shorter time to HIV rebound at the pre-ATI time point. The association between pre-ATI CD69+CD8+ T-cell and poor ATI viral outcomes was more robust compared with baseline with an agreement between with Spearman’s correlation analysis and Cox proportional model. CD69, a marker of T-cell activation and tissue memory T cells (32), was found negatively regulating the effector function of intra-tumoral T cells and was associated with CD8+ T-cell exhaustion (33, 34). CD69, together with PD-1 and CD103, was higher in the blood and tissue CD8+ T cells of PLWH than those without HIV, which was not reversible by ART (35). The inverse relationship between HIV rebound and circulating CD69+CD8+ T cells found in this study suggest that CD69+CD8+ T cells may represent cellular exhaustion or immune-cell dysfunction. To our knowledge, these findings have never been reported elsewhere (4, 7, 16, 28, 31) and will require further evaluation in larger studies.

Among glycomic biomarkers evaluated at baseline, those associated with poor ATI outcomes, reflected as shorter time to HIV rebound or smaller changes in intact proviral HIV-1 DNA, included fucosylated, non-galactosylated, non-sialylated, bisecting IgG N-glycans; galactosylated G1FB and G2FB species; IgG N-glycan G2; multiply-galactosylated glycans and sialylated glycans; GNA-binding glycans; NPA-binding glycans; WGA-binding glycans; and bisecting N-acetylglucosamine (GlcNAc) glycans; A3F1G3S3; and tri-galactosylated, FUC_A, and HB groups. In contrast, T/Tn-antigens (binding to MPA and HPA), non-galactosylated/non-sialylated glycans, and singly galactosylated and LB group glycans were associated with slightly improved ATI outcomes in our study. A2B and MPA-binding glycan associations were significant in both Spearman’s and Cox analyses. To our knowledge, GNA-binding, NPA-binding, and WGA-binding glycans have not been previously reported as potential HIV rebound biomarkers. GNA binds to Manα1-3Man mannose structures on HIV glycoprotein gp120 (36). NPA binds to Manα1-6Man glycans; these high-mannose glycans are ligands of dendritic cell receptors that internalize bound cargo and target it to antigen presentation pathways, fueling subsequent T-cell responses (37). WGA binds to sialic acid with a preference for α(2-3)-linked sialic acid residues, but also recognizes exposed GlcNAc residues (38). WGA binding is elevated in patients with myelodysplastic syndromes compared with healthy controls, and detects aberrantly increased glycan branching (39).

At the pre-ATI time point, most associations lost significance observed at baseline, including fucosylated IgG N-glycans and plasma glycans FA2BG0, FA2G0, and A3G3S3. The pre-ATI timepoint in our study was 1 day after the last vesatolimod dose; thus, this loss could be attributed to TLR7-mediated immune activation. A2B remained significant in both analyses at both time points, and GNA-binding glycans in both analyses pre-ATI.

Compared with pre-ATI glycomic biomarkers from studies in HIV non-controllers, some similarities were also found in our HIV controllers study. Baseline bisecting A2FB glycan trait in the IgG glycome correlated with shorter time to HIV rebound and shorter duration of suppression of HIV during ATI in our study, which aligned with results found in a pilot study of the Philadelphia cohort (28). Consistent with results from the Philadelphia and several AIDS Clinical Trials Group (ACTG) cohorts (4), associations between baseline plasma glycans FA2BG0 and FA2G0 and longer time to HIV rebound and A3G3S3 with shorter time to rebound were also found in this study. Increased galactosylation and decreased fucosylation enhance the ADCC of antibodies, whereas sialylation decreases the ADCC but enhances the anti-inflammatory activity of IgGs (40–43). Levels of plasma agalactosylated, core-fucosylated, non-sialylated IgG glycans were induced in HIV-positive unsuppressed individuals compared to both HIV-positive ART-suppressed and HIV-negative controls (44). Similarly, levels of IgG galactosylation and sialylation differentiated groups of people with chronic HIV suppressed on ART having more rapid rebound (with lower levels) vs. delayed rebound (with higher levels) during ATI (45). Together with our glycomic findings, this highlights the importance of circulating glycomes in modulating host inflammatory status and persistence of HIV.

Some of our glycomic findings differed from those of other HIV non-controller studies in the significance or in the direction of associations of several potential biomarkers, supporting the idea that this cohort is unique. PHA(E) lectin binds to complex N-glycans—preferring terminal Gal and GlcNAc glycans (46)—which are enriched in the HIV glycome (47). PHA(E)-binding glycans correlated with shorter time to HIV rebound in our study, but with longer time to rebound in the ACTG A5345 study (31). However, the complexity of the preferred PHA(E)-binding ligand may allow low-affinity binding of different glycans in a PHA(E) lectin-binding array as a limitation of the assay (48), contributing to discrepant results between these cohorts. In HIV controllers, higher baseline levels of HPA- and MPA-binding glycans were associated with longer time to HIV rebound and duration of control. HPA, deemed as a marker of poor cancer prognosis, selectively binds to alpha-GalNAc (α-GalNAc) and recognizes aberrant O-linked α-GlcNAc in cancer and metastasis-associated glycosylation changes (49, 50). MPA selectively binds to α-GalNAc, as well as Galβ1-3GalNAc (tumor-associated T-antigen glycans) and O-linked glycol peptides (51). MPA-binding glycans were associated with shorter time to viral rebound in cohorts with chronic HIV from the Philadelphia cohort and several ACTG clinical trials (4), but longer time to rebound in the ACTG A5345 study (31).

Baseline level of IgG N-glycan G2 in our study was correlated with poor viral outcomes. IgG N-glycan G2 is non-fucosylated and non-sialylated, both of which are associated with increased ADCC (42, 43), and has a terminal galactose, which induces ADCC (41). Pre-ATI levels of G2 were associated with longer time to viral rebound in ART-suppressed participants in the Philadelphia cohort and a panobinostat-treated cohort during ATI (4, 25, 28). G2 was also associated with plasmablast frequencies, an indicator of B-cell activation, in the panobinostat cohort (25). As mentioned earlier, these differences between studies may be attributed to different participant characteristics (HIV controllers on ART vs. non-controllers with chronic HIV suppressed on ART, and those with early-treated HIV), or TLR-7 mediated immune activation at pre-ATI. Additionally, HIV elite controllers, who are able to control HIV disease progression in the absence of ART, have higher levels of non-galactosylated HIV-specific antibodies, which are associated with more potent ADCC activity in this population (52).

Of all lipid/metabolite biomarkers evaluated, 14 baseline bioactive lipids had significant associations with better ATI viral outcomes in our cohort, including longer time to rebound and duration of control, and larger intact proviral HIV-1 DNA changes. These included glycoursodeoxycholic acid; trihexosylceramides; and species of phosphatidylcholine, phosphatidylinositol, cholesterol ester, ceramide, and sphingomyelin. Only three putative baseline lipid biomarkers were associated with poor ATI viral outcomes.

In the pre-ATI assessments of lipid/metabolite biomarkers, nine of them remained significant: trihexosylceramides, three species of phosphatidylcholine, ceramide (t17:1_24:0), cholesterol ester (20:0), and phosphatidylinositol retained their positive baseline associations with HIV control; and phosphatidylethanolamine and N6-acetyl-L-lysine retained their negative associations. However, the strong associations of glycoursodeoxycholic acid with all measures of HIV control and intact proviral HIV-1 DNA were not found in pre-ATI analyses in our study. This, along with the glycomic differences between baseline and pre-ATI assessments, suggest an effect of vesatolimod treatment other than changes in levels, as log_2_ fold-change was not significantly different between baseline and pre-ATI or between vesatolimod and placebo groups. To our knowledge, only one group—including members of this current team—has studied lipids and metabolites in the context of HIV viral dynamics during ATI in HIV non-controllers from Philadelphia and ACTG cohorts (4, 16). Consistent with the current findings in HIV controllers, glycoursodeoxycholic acid—a marker of bile acid metabolism—was associated with longer time to HIV rebound in the Philadelphia cohort (4).

Phosphatidylinositol and ceramide classes were associated with shorter time to HIV rebound in the Philadelphia cohort (16), in contrast with our population, where baseline levels of phosphatidylinositol species and ceramide (t17:1_24:0) were associated with improved ATI outcomes. Most of these were also significantly correlated pre-ATI. Ceramides are an extremely diverse group of lipids, with >1300 identified in human skin (53). Enhancement of long-chain ceramide levels in CD4+ T cells and macrophages has been shown to reduce HIV-1 infectivity *in vitro*, potentially through its effects on membrane microdomains (54). Although several groups of ceramides were associated with inflammation and immune activation or carotid artery plaque in HIV-1–infected individuals on ART, ceramide C24:0 (which includes ceramide [t17:1_24:0] from our study) was not (55). Further analysis will be required in order to determine whether the differences in ceramide findings are related to study population, ceramide species, or both. The phosphatidylinositol species that were significant here have not been previously reported in HIV biomarker studies, but phosphatidylinositols are reported to have anti-inflammatory activity (56). Polyunsaturated fatty acids (PUFAs), either free or as components of lipids such as phosphatidylinositol, have inhibitory effects on T-cell signaling and activation (57), which could contribute to our findings. In a cross-sectional study, plasma fatty acids differed between PLWH and healthy controls, with n-3, n-6, and n-9 PUFAs significantly lower in PLWH and were associated with advanced clinical disease (58). In particular, disturbed n-6 PUFA metabolism was associated with HIV replication and disease progression, which was not normalized to healthy control levels with ART (58). It is possible that actions of phosphatidylinositols in our HIV controller group are more similar to those in healthy individuals.

Several phosphatidylcholine species also significantly and positively correlated with pre-ATI CD4+ T cell–associated HIV DNA in the Philadelphia cohort (16), although, to our knowledge, a relationship between phosphatidylcholine species and duration of HIV control during ATI has not been published. In the current vesatolimod study, we found phosphatidylcholine species at baseline and pre-ATI that were associated with better ATI viral outcomes in the HIV controller population. However, baseline phosphatidylcholine (18:3_18:3) and phosphatidylethanolamine (19:1_18:1) were associated with poor ATI viral outcomes. Phosphatidylcholine has been reported to have anti-inflammatory activity (59, 60), but the roles of phosphatidylethanolamine in infection and disease appear complex (61–64). Some inconsistencies in our observations are potentially related to the physiologic complexity of lipid roles, including the potential for indirect pro-inflammatory actions (65). In addition, the composition of the lipidome shifts substantially with HIV ART in directions that depend on the specific regimen (66).

Additionally, vesatolimod treatment increased proinflammatory tryptophan metabolism, which was associated with shorter time to HIV rebound in previous work (4), and increased protein synthesis related to aminoacyl-tRNA biosynthesis. Diacylglycerols, lysophosphatidylcholine, lysophosphatidylinositol, phosphatidic acid, and phosphatidylserine were significantly upregulated by vesatolimod but not placebo between baseline and pre-ATI; lysophospholipids were also associated with shorter time to rebound in HIV non-controllers (16) and have pro-inflammatory activity (67, 68) in keeping with the vesatolimod MOA.

This study reports a positive relationship between proinflammatory biomarkers and poor viral outcomes. Host inflammatory pathways may be linked to exhaustion of effector CD8+ T cells and greater HIV replication, which may foster rebound during ATI even in HIV controllers. Interventions targeting sustained HIV suppression during ATI may require a balanced enhancement of innate and adaptive responses, which may only be obtained with combination treatments. Our study has several limitations. First the *post-hoc* nature of the analyses, the small number of participants, and restriction of the enrolled population narrowed the data interpretation and implications. Second, the data were skewed towards to those who received vesatolimod and accounted for two-thirds of the total study participants. Third, these analyses in HIV controllers were exploratory and preliminary because the data were not adjusted for potential confounding variables or multiple comparisons nor validated in another independent HIV controller cohort. There remains a knowledge gap in understanding glycome, lipidome and metabolome in HIV virology or persistence. The results require validation in larger independent cohorts, including those with and without a history of natural pre-ART viral control. Further work is also required to assess the prognostic and functional clinical significance of these putative biomarkers and address the associated MOA.

In conclusion, in this exploratory analysis we identified target host immunologic, glycomic, lipidomic, and metabolomic biomarkers that can be further evaluated as potential correlates of the duration of viral control post-ART cessation in HIV viremic controllers. We found previously unreported potential biomarkers—for example, CD69+CD8 T cells and the GNA-, NPA-, and WGA-binding glycans—that should be explored further. Disparities in the direction of associations we observed (e.g., IgG N-glycan G2, some phosphatidylinositol species and ceramide) between HIV controllers and the non-controller populations in other studies may be due to other factors or could represent a core difference in HIV biology between these populations that requires further study at both mechanistic and clinical levels.

## Data availability statement

The datasets presented in this article are not readily available because of data governance restrictions at Gilead Sciences. Requests to access the datasets should be directed to datarequest@gilead.com.

## Ethics statement

The studies involving humans were approved by Advarra IRB; and University of California, San Francisco. The studies were conducted in accordance with the local legislation and institutional requirements. The participants provided their written informed consent to participate in this study.

## Author contributions

MA-M: Conceptualization, Writing – original draft, Writing – review & editing, Formal analysis. SD: Formal analysis, Writing – original draft, Writing – review & editing, Data curation. LG: Formal analysis, Writing – original draft, Writing – review & editing. KH: Formal analysis, Writing – original draft, Writing – review & editing. AG: Formal analysis, Writing – original draft, Writing – review & editing. LZ: Formal analysis, Writing – original draft, Writing – review & editing. SH: Formal analysis, Writing – original draft, Writing – review & editing. DV: Formal analysis, Writing – original draft, Writing – review & editing. SG: Formal analysis, Writing – original draft, Writing – review & editing. LS: Formal analysis, Writing – original draft, Writing – review & editing. CdV: Formal analysis, Writing – original draft, Writing – review & editing. EV: Formal analysis, Writing – original draft, Writing – review & editing, Conceptualization. DS: Formal analysis, Writing – original draft, Writing – review & editing, Conceptualization. JW: Formal analysis, Writing – original draft, Writing – review & editing. YC: Formal analysis, Writing – original draft, Writing – review & editing, Conceptualization.
