# Supplementary material for: Circulating immune and plasma biomarkers of time to HIV rebound in HIV controllers treated with vesatolimod
Source: Front Immunol. 2024 Jun 24;15:1405348. doi: 10.3389/fimmu.2024.1405348 (PMC11229794; doi:10.3389/fimmu.2024.1405348)

**Data Supplement Table of Contents**

**Supplementary Table 1.** Association of baseline immune biomarkers with HIV control during ATI using Spearman’s analysis.

**Supplementary Table 2.** Association of baseline immune biomarkers with HIV control during ATI using Cox proportional hazards model.

**Supplementary Table 3.** Baseline glycomic biomarkers associated with HIV control during ATI using Spearman’s analysis.

**Supplementary Table 4.** Baseline glycomic biomarkers associated with HIV control during ATI using Cox proportional hazards model.

**Supplementary Table 5.** Baseline lipid/metabolite biomarkers associated with HIV control during ATI using Spearman’s analysis.

**Supplementary Table 6.** Association of pre-ATI immune biomarkers with HIV control during ATI using Spearman’s analysis.

**Supplementary Table 7.** Association of pre-ATI immune biomarkers with HIV control using Cox proportional hazards model.

**Supplementary Table 8.** A subset of pre-ATI glycomic biomarkers remained associated with HIV control during ATI.

**Supplementary Table 9.** A subset of pre-ATI glycomic biomarkers remained associated with HIV rebound during ATI using Cox proportional hazards model.

**Supplementary Table 10.** A subset of pre-ATI lipid/metabolite biomarkers remained associated with HIV control during ATI using Spearman’s analysis.

**Supplementary Table 11.** A subset of pre-ATI lipid/metabolite biomarkers remained associated with HIV control during ATI using Cox proportional hazards model.

**Supplementary Table 12.** Levels of most immune biomarkers were similar between vesatolimod and placebo at baseline and pre-ATI assessments (7/13-days post last dose).

**Supplementary Table 13.** No significant changes were observed in HIV rebound-associated glycomic biomarkers following vesatolimod treatment.

**Supplementary Table 14.** No significant changes were observed in HIV rebound-associated lipid/metabolite biomarkers following vesatolimod treatment.

**Supplementary Figure 1.** Protein synthesis–related pathways were associated with HIV rebound.

**Supplementary Figure 2.** Pro-inflammatory tryptophan metabolism and protein synthesis–related aminoacyl-tRNA biosynthesis were enriched after vesatolimod treatment.

**Supplementary Figure 3.** Vesatolimod mediated significant changes in more lipid subclasses than placebo.

**Supplementary Table 1.** Association of baseline immune biomarkers With HIV control during ATI using Spearman’s analysis. Data used for the analysis include the pooled vesatolimod and placebo groups and are limited to the analytes that pass quality control; for flow-based assays, cutoff is 70% viability. ATI, analytic treatment interruption; FDR, false discovery rate; GMFI, geometric mean fluorescent intensity; IFN, interferon; IP10, IFN γ-induced protein 10 kDa; ITAC, IFN-inducible T-cell alpha chemoattractant; NK, natural killer; pDC, plasmacytoid dendritic cells; pVL, plasma viral load.

|  |  | **Spearman’s Correlation** | | | | | | | | | | | |
| --- | --- | --- | --- | --- | --- | --- | --- | --- | --- | --- | --- | --- | --- |
|  | **N** | **Time to 1st Rebound to  Plasma HIV-1 RNA of 200 c/mL** | | | **Time to 1st Rebound to  Plasma HIV-1 RNA of 1000 c/mL** | | | **Time of Post-ATI**  **pVL ≤400 c/mL** | | | **Change in Intact Proviral HIV-1 DNA From Baseline** | | |
|  |  | **p-Value** | **R** | **FDR** | **p-Value** | **R** | **FDR** | **p-Value** | **R** | **FDR** | **p-Value** | **R** | **FDR** |
| CD14^+^CD16^+^ monocytes | 13 | 0.35 | 0.284 | 0.96 | 0.94 | -0.025 | 0.97 | 0.39 | 0.258 | 0.77 | 0.456 | 0.25 | 0.73 |
| CD16^+^ NK cells | 13 | 0.96 | -0.014 | 0.96 | 0.84 | -0.063 | 0.90 | 0.19 | -0.390 | 0.67 | 0.286 | -0.35 | 0.71 |
| CD8^+^CD38^+^ T cells | 13 | 0.94 | 0.025 | 0.96 | 0.29 | 0.320 | 0.76 | 0.36 | 0.275 | 0.77 | 0.838 | 0.07 | 0.93 |
| CD69^+^CD4^+^ T cells | 13 | 0.70 | -0.119 | 0.96 | 0.46 | -0.226 | 0.81 | 0.62 | -0.154 | 0.79 | 0.849 | -0.07 | 0.93 |
| CD69^+^CD8^+^ T cells | 13 | 0.05 | -0.549 | 0.53 | 0.02 | -0.642 | 0.32 | 0.20 | -0.379 | 0.67 | 0.108 | -0.51 | 0.71 |
| CD69^+^ NK cells | 13 | 0.92 | -0.030 | 0.96 | 0.73 | -0.105 | 0.85 | 0.36 | 0.275 | 0.77 | 0.892 | -0.05 | 0.93 |
| CD4^+^HLA-DR^+^CD38^+^ T cells | 13 | 0.63 | -0.146 | 0.96 | 0.53 | -0.190 | 0.85 | 0.53 | -0.192 | 0.77 | 0.229 | -0.40 | 0.71 |
| CD8^+^HLA-DR^+^CD38^+^ T cells | 13 | 0.42 | 0.243 | 0.96 | 0.48 | 0.218 | 0.81 | 0.13 | 0.445 | 0.67 | 0.527 | 0.21 | 0.77 |
| IFN-α | 21 | 0.80 | -0.058 | 0.96 | 0.67 | -0.098 | 0.85 | 0.46 | -0.171 | 0.77 | 0.419 | -0.20 | 0.71 |
| IL-1RA | 20 | 0.72 | 0.084 | 0.96 | 0.98 | 0.005 | 0.98 | 0.57 | -0.134 | 0.79 | 0.128 | -0.37 | 0.71 |
| IP10 | 21 | 0.40 | 0.193 | 0.96 | 0.06 | 0.422 | 0.64 | 0.24 | 0.268 | 0.67 | 0.728 | 0.09 | 0.93 |
| ITAC | 21 | 0.48 | 0.162 | 0.96 | 0.77 | -0.069 | 0.85 | 0.90 | -0.030 | 0.96 | 0.048 | 0.46 | 0.60 |
| CD4^+^Ki67^+^ T cells | 13 | 0.77 | 0.091 | 0.96 | 0.71 | 0.116 | 0.85 | 0.96 | -0.016 | 0.98 | 0.286 | -0.35 | 0.71 |
| CD8^+^Ki67^+^ T cells | 13 | 0.81 | 0.074 | 0.96 | 0.43 | 0.240 | 0.81 | 0.44 | 0.236 | 0.77 | 0.314 | -0.33 | 0.71 |
| pDC (CD40 GMFI) | 13 | 0.60 | -0.163 | 0.96 | 0.45 | -0.231 | 0.81 | 0.09 | -0.495 | 0.67 | 0.382 | -0.29 | 0.71 |
| pDC (CD54 GMFI) | 13 | 0.21 | 0.374 | 0.96 | 0.31 | 0.308 | 0.76 | 0.68 | 0.127 | 0.83 | 0.346 | 0.31 | 0.71 |

**Supplementary Table 2.** Association of baseline immune biomarkers with HIV control during ATI using Cox proportional hazards model. Data used for the analysis include the vesatolimod and placebo groups and are limited to the analytes that pass quality control; for flow-based assays, cutoff is 70% viability. ATI, analytic treatment interruption; CI, confidence interval; FDR, false discovery rate; GMFI, geometric mean fluorescent intensity; HR, hazard ratio; IFN, interferon; IP10, IFN γ-induced protein 10 kDa; ITAC, IFN-inducible T-cell alpha chemoattractant; NK, natural killer; pDC, plasmacytoid dendritic cells; pVL, plasma viral load.

|  | **n** | **Time to 1st Rebound to  Plasma HIV-1 RNA of 200 c/mL** | | | **Time to 1st Rebound to  Plasma HIV-1 RNA of 1000 c/mL** | | | **Time of Post-ATI**  **pVL ≤400 c/mL** | | |
| --- | --- | --- | --- | --- | --- | --- | --- | --- | --- | --- |
|  |  | **HR (95% CI)** | **p-Value** | **FDR** | **HR (95% CI)** | **p-Value** | **FDR** | **HR (95% CI)** | **p-Value** | **FDR** |
| CD14^+^CD16^+^ monocytes | 13 | 0.75 (0.24, 2.35) | 0.62 | 0.98 | 1.15 (0.35, 3.82) | 0.82 | 0.87 | 0.74 (0.23, 2.38) | 0.62 | 0.89 |
| CD16^+^ NK cells | 13 | 1.54 (0.47, 5.07) | 0.48 | 0.98 | 1.64 (0.49, 5.44) | 0.42 | 0.80 | 2.68 (0.74, 9.68) | 0.13 | 0.55 |
| CD38^+^CD8^+^ T cells | 13 | 1.28 (0.41, 4.03) | 0.67 | 0.98 | 0.78 (0.24, 2.47) | 0.67 | 0.80 | 0.63 (0.18, 2.17) | 0.46 | 0.89 |
| CD69^+^CD4^+^ T cells | 13 | 1.34 (0.42, 4.33) | 0.62 | 0.98 | 1.80 (0.56, 5.79) | 0.32 | 0.80 | 1.29 (0.41, 4.06) | 0.67 | 0.89 |
| CD69^+^CD8^+^ T cells | 13 | 2.24 (0.67, 7.53) | 0.19 | 0.98 | 2.60 (0.77, 8.81) | 0.13 | 0.80 | 1.19 (0.38, 3.73) | 0.76 | 0.89 |
| CD69^+^ NK cells | 13 | 1.49 (0.45, 5.00) | 0.52 | 0.98 | 0.80 (0.25, 2.55) | 0.70 | 0.80 | 0.37 (0.11, 1.29) | 0.12 | 0.55 |
| CD4^+^HLA-DR^+^CD38^+^ T cells | 13 | 2.57 (0.77, 8.58) | 0.12 | 0.98 | 2.42 (0.73, 8.04) | 0.15 | 0.80 | 3.17 (0.88, 11.45) | 0.08 | 0.55 |
| CD8^+^HLA-DR^+^CD38^+^ T cells | 13 | 1.83 (0.54, 6.19) | 0.33 | 0.98 | 1.69 (0.49, 5.76) | 0.40 | 0.80 | 1.01 (0.32, 3.17) | 0.98 | 0.98 |
| IFN-α | 21 | 0.55 (0.12, 2.45) | 0.43 | 0.98 | 0.58 (0.13, 2.6) | 0.47 | 0.80 | 0.62 (0.14, 2.82) | 0.53 | 0.89 |
| IL-1RA | 20 | 1.54 (0.58, 4.09) | 0.38 | 0.98 | 0.94 (0.38, 2.34) | 0.90 | 0.90 | 1.58 (0.62, 4.05) | 0.34 | 0.89 |
| IP10 | 21 | 1.05 (0.42, 2.62) | 0.92 | 0.98 | 0.71 (0.29, 1.73) | 0.45 | 0.80 | 0.77 (0.32, 1.87) | 0.57 | 0.89 |
| ITAC | 21 | 0.93 (0.39, 2.25) | 0.88 | 0.98 | 1.63 (0.64, 4.18) | 0.30 | 0.80 | 2.08 (0.77, 5.61) | 0.15 | 0.55 |
| CD4^+^Ki67^+^ T cells | 13 | 0.44 (0.11, 1.78) | 0.25 | 0.98 | 0.80 (0.25, 2.53) | 0.70 | 0.80 | 1.53 (0.46, 5.05) | 0.49 | 0.89 |
| CD8^+^Ki67^+^ T cells | 13 | 1.10 (0.35, 3.48) | 0.87 | 0.98 | 0.57 (0.18, 1.82) | 0.34 | 0.80 | 0.88 (0.28, 2.81) | 0.84 | 0.89 |
| pDC (CD40 GMFI) | 13 | 0.99 (0.32, 3.11) | 0.99 | 0.99 | 1.39 (0.42, 4.58) | 0.59 | 0.80 | 2.48 (0.67, 9.12) | 0.17 | 0.55 |
| pDC (CD54 GMFI) | 13 | 0.85 (0.27, 2.67) | 0.77 | 0.98 | 0.68 (0.21, 2.17) | 0.51 | 0.80 | 0.85 (0.26, 2.71) | 0.78 | 0.89 |

**Supplementary Table 3.** Baseline glycomic biomarkers associated with HIV control during ATI using Spearman’s analysis. Data include only participants with nominal p ≤0.05 for ≥1 plasma HIV-1 RNA copies/mL level in the pooled vesatolimod and placebo groups and are limited to the analytes that pass quality control. ATI, analytic treatment interruption; FDR, false discovery rate; FUC_A, antenna-fucosylated; GNA, *Galanthus nivalis* agglutinin; HB, high-branched; HPA, *Helix pomatia* agglutinin; IgG, immunoglobulin G; LB, low-branched; MPA, *Maclura pomifera* agglutinin; NPA, *Narcissus pseudonarcissus* agglutinin; PHA, *Phaseolus vulgaris* phytohemagglutinin.

|  |  | **Spearman’s Correlation** | | | | | | | | | | | |
| --- | --- | --- | --- | --- | --- | --- | --- | --- | --- | --- | --- | --- | --- |
| **Glycomic Biomarkers** | **n** | **Time to 1st Rebound to  Plasma HIV-1 RNA of 200 c/mL** | | | **Time to 1st Rebound to  Plasma HIV-1 RNA of 1000 c/mL** | | | **Duration of Plasma HIV-1 RNA  ≤400 c/mL During ATI** | | | **Change in Intact Proviral HIV-1 DNA From Baseline** | | |
|  |  | **p-Value** | **R** | **FDR** | **p-Value** | **R** | **FDR** | **p-Value** | **R** | **FDR** | **P-value** | **R** | **FDR** |
| **IgG N-glycans** |  |  |  |  |  |  |  |  |  |  |  |  |  |
| A2FB | 23 | 0.028 | -0.47 | 0.981 | 0.138 | -0.33 | 0.926 | 0.043 | -0.44 | 0.775 | 0.095 | -0.38 | 0.787 |
| A1FB | 23 | 0.029 | -0.47 | 0.981 | 0.166 | -0.31 | 0.926 | 0.126 | -0.34 | 0.775 | 0.069 | -0.41 | 0.787 |
| A2B | 23 | 0.246 | -0.26 | 0.981 | 0.180 | -0.3 | 0.926 | 0.048 | -0.43 | 0.775 | 0.315 | -0.24 | 0.858 |
| G2 | 23 | 0.824 | -0.05 | 0.981 | 0.984 | 0 | 0.990 | 0.827 | -0.05 | 0.998 | 0.017 | -0.53 | 0.695 |
| G2FB | 23 | 0.101 | -0.36 | 0.981 | 0.116 | -0.34 | 0.926 | 0.011 | -0.53 | 0.775 | 0.152 | -0.33 | 0.826 |
| **Plasma total glycans** |  |  |  |  |  |  |  |  |  |  |  |  |  |
| HPA-binding glycans | 23 | 0.005 | 0.57 | 0.981 | 0.001 | 0.64 | 0.247 | 0.016 | 0.5 | 0.775 | 0.305 | 0.24 | 0.858 |
| MPA-binding glycans | 23 | 0.067 | 0.39 | 0.981 | 0.041 | 0.43 | 0.926 | 0.019 | 0.49 | 0.775 | 0.971 | -0.01 | 0.995 |
| A3F1G3S3 | 23 | 0.637 | -0.11 | 0.981 | 0.317 | -0.22 | 0.932 | 0.751 | 0.07 | 0.998 | 0.032 | -0.47 | 0.695 |
| FA2BG0 | 23 | 0.503 | 0.15 | 0.981 | 0.037 | 0.45 | 0.926 | 0.359 | 0.21 | 0.998 | 0.603 | 0.12 | 0.910 |
| FA2G0 | 23 | 0.233 | 0.27 | 0.981 | 0.009 | 0.54 | 0.556 | 0.135 | 0.33 | 0.775 | 0.112 | 0.36 | 0.787 |
| FA2G1 | 23 | 0.444 | 0.17 | 0.981 | 0.196 | 0.29 | 0.926 | 0.782 | 0.06 | 0.998 | 0.039 | 0.45 | 0.695 |
| FUC_A_group | 23 | 0.621 | -0.11 | 0.981 | 0.325 | -0.22 | 0.932 | 0.743 | 0.07 | 0.998 | 0.032 | -0.47 | 0.695 |
| G total group | 23 | 0.231 | -0.27 | 0.981 | 0.008 | -0.55 | 0.556 | 0.114 | -0.35 | 0.775 | 0.192 | -0.30 | 0.826 |
| G0 group | 23 | 0.231 | 0.27 | 0.981 | 0.008 | 0.55 | 0.556 | 0.114 | 0.35 | 0.775 | 0.192 | 0.30 | 0.826 |
| G1_group | 23 | 0.514 | 0.15 | 0.981 | 0.244 | 0.26 | 0.926 | 0.770 | 0.07 | 0.998 | 0.038 | 0.46 | 0.695 |
| G3 group | 23 | 0.306 | -0.23 | 0.981 | 0.114 | -0.35 | 0.926 | 0.872 | 0.04 | 0.998 | 0.031 | -0.47 | 0.695 |
| GNA-binding glycans | 23 | 0.056 | -0.40 | 0.981 | 0.645 | -0.10 | 0.937 | 0.040 | -0.43 | 0.775 | 0.486 | -0.16 | 0.858 |
| HB group | 23 | 0.572 | -0.13 | 0.981 | 0.420 | -0.18 | 0.932 | 0.518 | 0.15 | 0.998 | 0.025 | -0.49 | 0.695 |
| LB group | 23 | 0.572 | 0.13 | 0.981 | 0.420 | 0.18 | 0.932 | 0.518 | -0.15 | 0.998 | 0.025 | 0.49 | 0.695 |
| PHA(E)-binding glycans | 23 | 0.035 | -0.44 | 0.981 | 0.211 | -0.27 | 0.926 | 0.969 | -0.01 | 0.998 | 0.996 | 0 | 1.000 |
| S total group | 23 | 0.312 | -0.23 | 0.981 | 0.045 | -0.43 | 0.926 | 0.421 | -0.18 | 0.998 | 0.100 | -0.37 | 0.787 |
| S0 group | 23 | 0.312 | 0.23 | 0.981 | 0.045 | 0.43 | 0.926 | 0.421 | 0.18 | 0.998 | 0.100 | 0.37 | 0.787 |

**Supplementary Table 4.** Baseline glycomic biomarkers associated with HIV control during ATI using Cox proportional hazards model. Data used for the analysis include the vesatolimod and placebo groups and are limited to the analytes that pass quality control. Data include only participants with nominal p ≤0.05 for ≥1 plasma HIV-1 RNA copies/mL level. ATI, analytic treatment interruption; FDR, false discovery rate; GSL, *griffania simplicifolia* lectin; HR hazard ratio; MPA, *Maclura pomifera* agglutinin; NPA, *Narcissus pseudonarcissus* agglutinin; WGA, wheat-germ agglutinin.

|  |  | **Cox Proportional Hazards Model** | | | | | | | | |
| --- | --- | --- | --- | --- | --- | --- | --- | --- | --- | --- |
| **Glycomic Biomarkers** |  | **Time to 1^st^ Rebound to  Plasma HIV-1 RNA of 200 c/mL** | | | **Time to 1^st^ Rebound to  Plasma HIV-1 RNA of 1000 c/mL** | | | **Duration of Plasma HIV-1 RNA  ≤400 c/mL During ATI** | | |
|  | **n** | **HR (95% CI)** | **p-Value** | **FDR** | **HR (95% CI)** | **p-Value** | **FDR** | **HR (95% CI)** | **p-Value** | **FDR** |
| **IgG N-glycans** |  |  |  |  |  |  |  |  |  |  |
| A2B | 22 | 1.3 (0.5, 3.2) | 0.540 | 0.969 | 1.9 (0.7, 4.8) | 0.180 | 0.984 | 4.3 (1.4, 13.4) | 0.013 | 0.991 |
| G1FB | 22 | 2.6 (1.0, 7.0) | 0.050 | 0.969 | 1.9 (0.8, 4.6) | 0.170 | 0.984 | 2.4 (1.0, 6.1) | 0.060 | 0.991 |
| **Plasma total glycans** |  |  |  |  |  |  |  |  |  |  |
| A3G3S3 | 22 | 3.3 (1.1, 10.4) | 0.037 | 0.969 | 3.6 (1.1, 11.1) | 0.028 | 0.984 | 1.7 (0.6, 4.8) | 0.320 | 0.991 |
| G2 group | 22 | 1.6 (0.7, 3.7) | 0.310 | 0.969 | 2.7 (1.1, 6.8) | 0.036 | 0.984 | 3.0 (1.1, 8.0) | 0.029 | 0.991 |
| GSL II | 23 | 1.9 (0.8, 4.5) | 0.160 | 0.969 | 3.0 (1.1, 7.9) | 0.029 | 0.984 | 2.2 (0.9, 5.3) | 0.090 | 0.991 |
| MPA-binding glycans | 23 | 0.4 (0.1, 0.9) | 0.021 | 0.969 | 0.2 (0.1, 0.6) | 0.004 | 0.890 | 0.2 (0.1, 0.6) | 0.004 | 0.991 |
| NPA-binding glycans | 23 | 2.2 (0.9, 5.2) | 0.090 | 0.969 | 1.2 (0.5, 2.8) | 0.640 | 0.984 | 2.4 (1.0, 5.9) | 0.050 | 0.991 |
| WGA-binding glycans | 23 | 3.5 (1.3, 9.4) | 0.011 | 0.969 | 2.4 (1.0, 5.7) | 0.050 | 0.984 | 1.6 (0.7, 3.7) | 0.300 | 0.991 |

**Supplementary Table 5.** Baseline lipid/metabolite biomarkers associated with HIV control during ATI using Spearman’s analysis. Data include participants with nominal p ≤0.05 for ≥3 viral outcomes in the pooled vesatolimod and placebo groups and are limited to the analytes that pass quality control. ATI, analytic treatment interruption; FDR, false discovery rate.

|  |  | **Spearman’s Correlation** | | | | | | | | | | | |
| --- | --- | --- | --- | --- | --- | --- | --- | --- | --- | --- | --- | --- | --- |
| **Biomarkers** | **n** | **Time to 1st Rebound to  Plasma HIV-1 RNA of 200 c/mL** | | | **Time to 1st Rebound to  Plasma HIV-1 RNA of 1000 c/mL** | | | **Duration of Plasma HIV-1 RNA  ≤400 c/mL During ATI** | | | **Change in Intact Proviral  HIV-1 DNA From Baseline** | | |
|  |  | **p-Value** | **R** | **FDR** | **p-Value** | **R** | **FDR** | **p-Value** | **R** | **FDR** | p-Value | R | FDR |
| Glycoursodeoxycholic acid | 22 | 0.017 | 0.50 | 0.680 | 0.001 | 0.67 | 0.280 | 0.007 | 0.57 | 0.954 | 0.041 | 0.45 | 0.994 |
| Phosphatidylcholine (16:2e_12:0) | 22 | 0.011 | 0.53 | 0.999 | 0.050 | 0.42 | 0.998 | 0.127 | 0.34 | 0.912 | 0.001 | 0.67 | 0.868 |
| Ceramide (t17:1_24:0) | 22 | 0.006 | 0.57 | 0.999 | 0.046 | 0.43 | 0.998 | 0.132 | 0.33 | 0.915 | 0.010 | 0.55 | 0.937 |
| Sphingomyelin (d28:0) | 22 | 0.030 | 0.46 | 0.999 | 0.018 | 0.50 | 0.998 | 0.062 | 0.41 | 0.887 | 0.014 | 0.53 | 0.937 |
| Phosphatidylinositol (19:0_18:2) | 22 | 0.031 | 0.46 | 0.999 | 0.225 | 0.27 | 0.998 | 0.016 | 0.51 | 0.887 | 0.015 | 0.52 | 0.937 |
| Phosphatidylinositol (18:0e_20:4) | 22 | 0.038 | 0.45 | 0.999 | 0.093 | 0.37 | 0.998 | 0.028 | 0.47 | 0.887 | 0.030 | 0.47 | 0.937 |
| Phosphatidylcholine (16:0_16:0) | 22 | 0.017 | 0.50 | 0.999 | 0.022 | 0.49 | 0.998 | 0.171 | 0.30 | 0.953 | 0.039 | 0.45 | 0.937 |
| Phosphatidylcholine (20:4_20:4) | 22 | 0.038 | 0.45 | 0.999 | 0.288 | 0.24 | 0.998 | 0.011 | 0.53 | 0.887 | 0.041 | 0.45 | 0.937 |
| Phosphatidylinositol (18:0_18:0) | 22 | 0.033 | 0.46 | 0.999 | 0.030 | 0.46 | 0.998 | 0.022 | 0.49 | 0.887 | 0.224 | 0.28 | 0.937 |
| N6-acetyl-L-lysine | 22 | 0.006 | -0.56 | 0.680 | 0.012 | -0.53 | 0.836 | 0.013 | -0.53 | 0.954 | 0.655 | -0.10 | 0.994 |

**Supplementary Table 6.** Association of pre-ATI immune biomarkers with HIV control during ATI using Spearman’s analysis. Data used for the analysis include the vesatolimod and placebo groups and are limited to the analytes that pass quality control. ATI, analytic treatment interruption; FDR, false discovery rate; GMFI, geometric mean fluorescent intensity; IFN, interferon; IP10, IFN γ-induced protein 10 kDa; ITAC, IFN-inducible T-cell alpha chemoattractant; NK, natural killer; pDC, plasmacytoid dendritic cells; pVL, plasma viral load.

|  |  | **Spearman’s Correlation** | | | | | | | | | | | |
| --- | --- | --- | --- | --- | --- | --- | --- | --- | --- | --- | --- | --- | --- |
| **Immune biomarkers** | **N** | **Time to 1^st^ Rebound to  Plasma HIV-1 RNA of 200 c/mL** | | | **Time to 1^st^ Rebound to  Plasma HIV-1 RNA of 1000 c/mL** | | | **Time of Post-ATI**  **pVL ≤400 c/mL** | | | **Change in Intact Proviral HIV-1 DNA From Baseline** | | |
|  |  | **p-Value** | **R** | **FDR** | **p-Value** | **R** | **FDR** | **p-Value** | **R** | **FDR** | **p-Value** | **R** | **FDR** |
| CD14^+^CD16^+^ monocytes | 21 | 0.280 | 0.249 | 0.96 | 0.3000 | 0.239 | 0.76 | 0.980 | -0.005 | 0.98 | 0.615 | 0.12 | 0.856 |
| CD16^+^ NK cells | 21 | 0.200 | -0.289 | 0.96 | 0.3000 | -0.237 | 0.76 | 0.390 | -0.197 | 0.77 | 0.383 | -0.21 | 0.709 |
| CD38^+^CD8^+^ T cells | 21 | 0.610 | 0.119 | 0.96 | 0.2600 | 0.258 | 0.76 | 0.610 | 0.117 | 0.79 | 0.197 | 0.31 | 0.709 |
| CD69^+^CD4^+^ T cells | 21 | 0.270 | -0.252 | 0.96 | 0.6000 | -0.120 | 0.85 | 0.150 | -0.322 | 0.67 | 0.204 | -0.30 | 0.709 |
| CD69^+^CD8^+^ T cells | 21 | 0.001 | -0.685 | 0.02 | 0.0004 | -0.698 | 0.01 | 0.007 | -0.581 | 0.22 | 0.004 | -0.63 | 0.128 |
| CD69^+^ NK cells | 21 | 0.150 | -0.322 | 0.96 | 0.2600 | -0.258 | 0.76 | 0.170 | -0.313 | 0.67 | 0.643 | -0.11 | 0.857 |
| CD4^+^HLA-DR^+^CD38^+^ T cells | 21 | 0.930 | -0.021 | 0.96 | 0.3100 | -0.232 | 0.76 | 0.110 | -0.356 | 0.67 | 0.963 | -0.01 | 0.963 |
| CD8^+^HLA-DR^+^CD38^+^ T cells | 21 | 0.940 | 0.016 | 0.96 | 0.7300 | -0.081 | 0.85 | 0.430 | -0.183 | 0.77 | 0.872 | 0.04 | 0.925 |
| IFN-α | 23 | 0.590 | -0.118 | 0.96 | 0.1800 | -0.292 | 0.76 | 0.250 | -0.251 | 0.67 | 0.386 | -0.20 | 0.709 |
| IL-1RA | 20 | 0.960 | 0.013 | 0.96 | 0.7700 | 0.069 | 0.85 | 0.880 | -0.038 | 0.96 | 0.421 | 0.20 | 0.709 |
| IP10 | 23 | 0.240 | 0.253 | 0.96 | 0.4100 | 0.180 | 0.81 | 0.720 | 0.080 | 0.83 | 0.790 | 0.06 | 0.925 |
| ITAC | 23 | 0.650 | 0.100 | 0.96 | 0.7200 | -0.079 | 0.85 | 0.730 | -0.075 | 0.83 | 0.056 | 0.42 | 0.597 |
| CD4^+^Ki67^+^ T cells | 21 | 0.870 | -0.038 | 0.96 | 0.4000 | -0.195 | 0.81 | 0.220 | -0.277 | 0.67 | 0.519 | -0.16 | 0.767 |
| CD8^+^Ki67^+^ T cells | 21 | 0.510 | -0.152 | 0.96 | 0.6000 | -0.120 | 0.85 | 0.520 | -0.149 | 0.77 | 0.383 | -0.21 | 0.709 |
| pDC (CD40 GMFI) | 21 | 0.700 | -0.090 | 0.96 | 0.2500 | -0.260 | 0.76 | 0.090 | -0.384 | 0.67 | 0.411 | -0.20 | 0.709 |
| pDC (CD54 GMFI) | 21 | 0.050 | 0.432 | 0.53 | 0.2500 | 0.261 | 0.76 | 0.530 | 0.146 | 0.77 | 0.896 | 0.03 | 0.925 |

**Supplementary Table 7.** Association of pre-ATI immune biomarkers with HIV control using Cox proportional hazards model. Data used for the analysis include the vesatolimod and placebo groups and are limited to the analytes that pass quality control. ATI, analytic treatment interruption; CI, confidence interval; FDR, false discovery rate; GMFI, geometric mean fluorescent intensity; HR, hazard ratio; IFN, interferon; IP10, IFN γ-induced protein 10 kDa; ITAC, IFN-inducible T-cell alpha chemoattractant; NK, natural killer; pDC, plasmacytoid dendritic cells; pVL, plasma viral load.

|  | **n** | **Time to 1st Rebound to  Plasma HIV-1 RNA of 200 c/mL** | | | **Time to 1st Rebound to  Plasma HIV-1 RNA of 1000 c/mL** | | | **Time of Post-ATI**  **pVL ≤400 c/mL** | | |
| --- | --- | --- | --- | --- | --- | --- | --- | --- | --- | --- |
|  |  | **HR (95% CI)** | **p-Value** | **FDR** | **HR (95% CI)** | **p-Value** | **FDR** | **HR (95% CI)** | **p-Value** | **FDR** |
| CD14^+^CD16^+^ monocytes | 21 | 0.60 (0.24, 1.47) | 0.260 | 0.67 | 0.58 (0.24, 1.44) | 0.240 | 0.580 | 1.48 (0.59, 3.72) | 0.410 | 0.720 |
| CD16^+^ NK cells | 21 | 1.39 (0.57, 3.41) | 0.470 | 0.75 | 1.68 (0.67, 4.2) | 0.270 | 0.580 | 1.12 (0.46, 2.71) | 0.810 | 0.810 |
| CD38^+^CD8^+^ T cells | 21 | 0.60 (0.24, 1.46) | 0.260 | 0.67 | 0.75 (0.31, 1.81) | 0.520 | 0.820 | 0.88 (0.36, 2.12) | 0.770 | 0.810 |
| CD69^+^CD4^+^ T cells | 21 | 1.12 (0.46, 2.73) | 0.800 | 0.91 | 0.58 (0.23, 1.48) | 0.260 | 0.580 | 1.15 (0.48, 2.78) | 0.750 | 0.810 |
| CD69^+^CD8^+^ T cells | 21 | 10.81 (2.22, 52.57) | 0.003 | 0.05 | 7.93 (2.08, 30.19) | 0.002 | 0.039 | 3.88 (1.41, 10.71) | 0.009 | 0.141 |
| CD69^+^ NK cells | 21 | 1.70 (0.69, 4.16) | 0.250 | 0.67 | 1.19 (0.49, 2.91) | 0.700 | 0.820 | 1.2 (0.49, 2.95) | 0.700 | 0.810 |
| CD4^+^HLA-DR^+^CD38^+^ T cells | 21 | 0.58 (0.21, 1.6) | 0.290 | 0.67 | 1.48 (0.59, 3.67) | 0.400 | 0.710 | 2.44 (0.91, 6.53) | 0.080 | 0.410 |
| CD8^+^HLA-DR^+^CD38^+^ T cells | 21 | 1.13 (0.46, 2.74) | 0.790 | 0.91 | 1.70 (0.67, 4.35) | 0.260 | 0.580 | 1.53 (0.62, 3.82) | 0.360 | 0.710 |
| IFN-α | 23 | 2.14 (0.46, 9.98) | 0.330 | 0.67 | 4.70 (0.89, 24.83) | 0.070 | 0.550 | 3.59 (0.74, 17.44) | 0.110 | 0.450 |
| IL-1RA | 20 | 1.26 (0.51, 3.13) | 0.620 | 0.90 | 1.06 (0.43, 2.62) | 0.910 | 0.970 | 1.65 (0.64, 4.27) | 0.30 | 0.690 |
| IP10 | 23 | 0.68 (0.29, 1.62) | 0.390 | 0.69 | 0.82 (0.35, 1.93) | 0.660 | 0.820 | 1.29 (0.53, 3.16) | 0.570 | 0.810 |
| ITAC | 23 | 1.07 (0.45, 2.53) | 0.880 | 0.94 | 1.17 (0.50, 2.72) | 0.720 | 0.820 | 2.08 (0.78, 5.51) | 0.140 | 0.460 |
| CD4^+^Ki67^+^ T cells | 21 | 0.61 (0.24, 1.52) | 0.280 | 0.67 | 1.24 (0.50, 3.09) | 0.640 | 0.820 | 1.64 (0.65, 4.13) | 0.300 | 0.690 |
| CD8^+^Ki67^+^ T cells | 21 | 1.03 (0.42, 2.52) | 0.950 | 0.95 | 1.02 (0.42, 2.46) | 0.970 | 0.970 | 1.2 (0.49, 2.92) | 0.690 | 0.810 |
| pDC (CD40 GMFI) | 21 | 1.13 (0.46, 2.74) | 0.790 | 0.91 | 1.66 (0.65, 4.25) | 0.290 | 0.580 | 2.32 (0.92, 5.87) | 0.070 | 0.410 |
| pDC (CD54 GMFI) | 21 | 0.63 (0.26, 1.55) | 0.320 | 0.67 | 0.52 (0.21, 1.32) | 0.170 | 0.580 | 0.82 (0.34, 2.00) | 0.670 | 0.810 |

**Supplementary Table 8.** A subset of pre-ATI glycomic biomarkers remained associated with HIV control during ATI. Data used for the analysis include the pooled vesatolimod and placebo arms and are limited to the analytes that pass quality control. Nominal p-values are presented. ATI, analytic treatment interruption; FDR, false discovery rate; FUC_A, antenna-fucosylated; GNA, *Galanthus nivalis* agglutinin; GSL, *Griffania simplicifolia* lectin; IgG, immunoglobulin G; HB, high-branched; HPA, *Helix pomatia* agglutinin; LB, low-branched; MPA, *Maclura pomifera* agglutinin; NPA, *Narcissus pseudonarcissus* agglutinin; PHA, *Phaseolus vulgaris* phytohaemagglutinin.

| **Biomarkers** | | **n** | **Spearman’s Correlation** | | | | | | | | | | | |
| --- | --- | --- | --- | --- | --- | --- | --- | --- | --- | --- | --- | --- | --- | --- |
|  |  |  | **Time to 1st Rebound to  Plasma HIV-1 RNA of  200 c/mL** | | | **Time to 1st Rebound to  Plasma HIV-1 RNA of  1000 c/mL** | | | **Duration of Plasma HIV-1 RNA  ≤400 c/mL During ATI** | | | **Change in Intact Proviral HIV-1 DNA From Baseline** | | |
|  |  |  | **p-Value** | **R** | **FDR** | **p-Value** | **R** | **FDR** | **p-Value** | **R** | **FDR** | **P-value** | **R** | **FDR** |
| **IgG**  **N-glycans** | A1FB | 23 | 0.147 | -0.31 | 0.981 | 0.497 | -0.15 | 0.932 | 0.604 | -0.11 | 0.998 | 0.192 | -0.30 | 0.826 |
|  | A2B | 23 | 0.241 | -0.25 | 0.981 | 0.182 | -0.29 | 0.926 | 0.036 | -0.44 | 0.775 | 0.276 | -0.25 | 0.858 |
|  | A2FB | 23 | 0.204 | -0.28 | 0.981 | 0.428 | -0.17 | 0.932 | 0.165 | -0.30 | 0.854 | 0.261 | -0.26 | 0.858 |
|  | G2 | 23 | 0.485 | -0.15 | 0.981 | 0.421 | -0.18 | 0.932 | 0.916 | -0.02 | 0.998 | 0.045 | -0.44 | 0.695 |
|  | G2FB | 23 | 0.276 | -0.24 | 0.981 | 0.463 | -0.16 | 0.932 | 0.260 | -0.24 | 0.998 | 0.179 | -0.30 | 0.826 |
| **Plasma total glycans** | A3F1G3S3 | 23 | 0.494 | -0.15 | 0.981 | 0.545 | -0.13 | 0.932 | 0.968 | -0.01 | 0.998 | 0.088 | -0.38 | 0.787 |
|  | A3G3S3 | 23 | 0.544 | -0.13 | 0.981 | 0.578 | -0.12 | 0.932 | 0.732 | -0.08 | 0.998 | 0.885 | -0.03 | 0.979 |
|  | FA2BG0 | 23 | 0.834 | -0.05 | 0.981 | 0.478 | 0.16 | 0.932 | 0.631 | 0.11 | 0.998 | 0.720 | -0.08 | 0.931 |
|  | FA2G0 | 23 | 0.901 | 0.03 | 0.981 | 0.436 | 0.17 | 0.932 | 0.449 | 0.17 | 0.998 | 0.414 | 0.19 | 0.858 |
|  | FA2G1 | 23 | 0.405 | -0.18 | 0.981 | 0.819 | -0.05 | 0.960 | 0.764 | -0.07 | 0.998 | 0.152 | 0.32 | 0.826 |
|  | FUC_A group | 23 | 0.492 | -0.15 | 0.981 | 0.490 | -0.15 | 0.932 | 0.873 | 0.04 | 0.998 | 0.046 | -0.44 | 0.695 |
|  | G Total group | 23 | 0.946 | -0.01 | 0.981 | 0.447 | -0.17 | 0.932 | 0.478 | -0.16 | 0.998 | 0.511 | -0.15 | 0.858 |
|  | G0 group | 23 | 0.946 | 0.01 | 0.981 | 0.447 | 0.17 | 0.932 | 0.478 | 0.16 | 0.998 | 0.511 | 0.15 | 0.858 |
|  | G1 group | 23 | 0.360 | -0.20 | 0.981 | 0.640 | -0.10 | 0.937 | 0.764 | -0.07 | 0.998 | 0.198 | 0.29 | 0.826 |
|  | G2 group | 23 | 0.428 | 0.17 | 0.981 | 0.736 | 0.07 | 0.950 | 0.716 | -0.08 | 0.998 | 0.310 | 0.23 | 0.858 |
|  | G3 group | 23 | 0.242 | -0.25 | 0.981 | 0.361 | -0.20 | 0.932 | 0.804 | -0.05 | 0.998 | 0.013 | -0.53 | 0.695 |
|  | GNA-binding glycan | 23 | 0.128 | -0.33 | 0.981 | 0.459 | -0.16 | 0.932 | 0.025 | -0.47 | 0.775 | 0.383 | -0.20 | 0.858 |
|  | GSL II | 23 | 0.492 | 0.15 | 0.981 | 0.841 | 0.04 | 0.960 | 0.900 | 0.03 | 0.998 | 0.600 | -0.12 | 0.910 |
|  | HB group | 23 | 0.328 | -0.21 | 0.981 | 0.363 | -0.20 | 0.932 | 0.873 | -0.04 | 0.998 | 0.039 | -0.45 | 0.695 |
|  | HPA-binding glycan | 23 | 0.324 | -0.22 | 0.981 | 0.343 | -0.21 | 0.932 | 0.060 | -0.40 | 0.775 | 0.445 | -0.18 | 0.858 |
|  | LB group | 23 | 0.328 | 0.21 | 0.981 | 0.363 | 0.20 | 0.932 | 0.873 | 0.04 | 0.998 | 0.039 | 0.45 | 0.695 |
|  | MPA-binding glycan | 23 | 0.252 | -0.25 | 0.981 | 0.277 | -0.24 | 0.926 | 0.529 | -0.14 | 0.998 | 0.207 | -0.29 | 0.833 |
|  | NPA-binding glycan | 23 | 0.934 | -0.02 | 0.981 | 0.242 | 0.25 | 0.926 | 0.948 | -0.01 | 0.998 | 0.969 | 0.01 | 0.995 |
|  | PHA(E)-binding glycan | 23 | 0.588 | 0.12 | 0.981 | 0.661 | -0.10 | 0.939 | 0.998 | 0 | 0.998 | 0.429 | -0.18 | 0.858 |
|  | S Total group | 23 | 0.154 | 0.31 | 0.981 | 0.428 | 0.17 | 0.932 | 0.601 | 0.11 | 0.998 | 0.486 | -0.16 | 0.858 |
|  | S0 group | 23 | 0.154 | -0.31 | 0.981 | 0.428 | -0.17 | 0.932 | 0.601 | -0.11 | 0.998 | 0.486 | 0.16 | 0.858 |
|  | WGA-binding glycan | 23 | 0.141 | -0.32 | 0.981 | 0.070 | -0.38 | 0.926 | 0.052 | -0.41 | 0.775 | 0.364 | -0.21 | 0.858 |

**Supplementary Table 9.** A subset of pre-ATI glycomic biomarkers remained associated with HIV rebound during ATI using Cox proportional hazards model. Data used for the analysis include the vesatolimod and placebo groups and are limited to the analytes that pass quality control. Data include only participants with nominal p ≤0.05 for ≥1 plasma HIV-1 RNA c/mL level. ATI, analytic treatment interruption; CI, confidence interval; FDR, false discovery rate; FUC_A, antenna-fucosylated; GSL, *griffania simplicifolia* lectin; GNA, *Galanthus nivalis* agglutinin; HR, hazard ratio; IgG, immunoglobulin G; HPA, *Helix pomatia* agglutinin; MPA, *Maclura pomifera* agglutinin; NPA, *Narcissus pseudonarcissus* agglutinin; PHA, *Phaseolus vulgaris* phytohaemagglutinin.

| Biomarkers | | n | **Cox Proportional Hazards Model** | | | | | | | | |
| --- | --- | --- | --- | --- | --- | --- | --- | --- | --- | --- | --- |
|  |  |  | **Time to 1st Rebound to  Plasma HIV-1 RNA of 200 c/mL** | | | Time to 1st Rebound to  Plasma HIV-1 RNA of 1000 c/mL | | | **Duration of Plasma HIV-1 RNA  ≤400 c/mL During ATI** | | |
|  |  |  | **HR (95% CI)** | **p-Value** | **FDR** | **HR (95% CI)** | **p-Value** | **FDR** | **HR (95% CI)** | **p-Value** | **FDR** |
| **IgG**  **N-glycans** | A1FB | 23 | 1.2 (0.5, 2.9) | 0.653 | 0.969 | 0.9 (0.4, 2.1) | 0.792 | 0.984 | 0.7 (0.3, 1.7) | 0.423 | 0.991 |
|  | A2B | 23 | 1.3 (0.5, 3.0) | 0.595 | 0.969 | 1.9 (0.7, 4.7) | 0.178 | 0.984 | 2.5 (1.0, 6.4) | 0.050 | 0.991 |
|  | A2FB | 23 | 1.4 (0.6, 3.2) | 0.464 | 0.969 | 1.0 (0.4, 2.4) | 0.929 | 0.984 | 1.2 (0.5, 2.8) | 0.668 | 0.991 |
|  | G2 | 23 | 1.4 (0.6, 3.3) | 0.424 | 0.969 | 1.4 (0.6, 3.4) | 0.404 | 0.984 | 0.9 (0.4, 2.1) | 0.771 | 0.991 |
|  | G2FB | 23 | 1.0 (0.4, 2.3) | 0.924 | 0.995 | 0.8 (0.4, 2.0) | 0.704 | 0.984 | 1.1 (0.5, 2.6) | 0.809 | 0.991 |
| **Plasma total glycans** | A3F1G3S3 | 23 | 1.1 (0.5, 2.5) | 0.870 | 0.985 | 1.1 (0.5, 2.5) | 0.866 | 0.984 | 0.7 (0.3, 1.7) | 0.423 | 0.991 |
|  | A3G3S3 | 23 | 1.4 (0.5, 3.7) | 0.473 | 0.969 | 1.3 (0.5, 3.3) | 0.611 | 0.984 | 1.2 (0.5, 3.1) | 0.737 | 0.991 |
|  | FA2BG0 | 23 | 1.6 (0.7, 3.7) | 0.317 | 0.969 | 0.9 (0.4, 2.2) | 0.878 | 0.984 | 1.1 (0.5, 2.7) | 0.751 | 0.991 |
|  | FA2G0 | 23 | 1.3 (0.5, 3.1) | 0.576 | 0.969 | 1.2 (0.5, 2.7) | 0.737 | 0.984 | 1.1 (0.5, 2.6) | 0.774 | 0.991 |
|  | FA2G1 | 23 | 1.7 (0.7, 3.9) | 0.246 | 0.969 | 1.8 (0.7, 4.2) | 0.206 | 0.984 | 1.2 (0.5, 2.8) | 0.646 | 0.991 |
|  | FUC_A group | 23 | 1.1 (0.5, 2.5) | 0.870 | 0.985 | 1.1 (0.5, 2.5) | 0.866 | 0.984 | 0.7 (0.3, 1.7) | 0.423 | 0.991 |
|  | G Total group | 23 | 0.7 (0.3, 1.8) | 0.505 | 0.969 | 0.8 (0.4, 2.0) | 0.705 | 0.984 | 0.8 (0.3, 1.8) | 0.590 | 0.991 |
|  | G0 group | 23 | 1.3 (0.5, 3.1) | 0.576 | 0.969 | 1.2 (0.5, 2.7) | 0.737 | 0.984 | 1.1 (0.5, 2.6) | 0.774 | 0.991 |
|  | G1 group | 23 | 1.7 (0.7, 3.9) | 0.246 | 0.969 | 1.8 (0.7, 4.2) | 0.206 | 0.984 | 1.2 (0.5, 2.8) | 0.646 | 0.991 |
|  | G2 group | 23 | 0.8 (0.4, 2.0) | 0.687 | 0.969 | 0.8 (0.3, 1.9) | 0.621 | 0.984 | 1.3 (0.6, 3.2) | 0.518 | 0.991 |
|  | G3 group | 23 | 1.5 (0.6, 3.4) | 0.395 | 0.969 | 1.5 (0.6, 3.5) | 0.385 | 0.984 | 0.8 (0.3, 2.0) | 0.675 | 0.991 |
|  | GNA-binding glycan | 23 | 1.5 (0.6, 3.5) | 0.360 | 0.969 | 1.6 (0.7, 3.7) | 0.305 | 0.984 | 2.5 (1.1, 6.2) | 0.038 | 0.991 |
|  | GSL II | 23 | 0.8 (0.3, 1.8) | 0.512 | 0.969 | 0.8 (0.3, 2.0) | 0.650 | 0.984 | 1.1 (0.5, 2.5) | 0.855 | 0.991 |
|  | HB group | 23 | 1.1 (0.5, 2.5) | 0.870 | 0.985 | 1.1 (0.5, 2.5) | 0.866 | 0.984 | 0.7 (0.3, 1.7) | 0.423 | 0.991 |
|  | HPA-binding glycan | 23 | 1.6 (0.7, 3.8) | 0.299 | 0.969 | 1.6 (0.7, 3.9) | 0.278 | 0.984 | 2.3 (0.9, 5.5) | 0.066 | 0.991 |
|  | LB group | 23 | 0.8 (0.3, 1.9) | 0.590 | 0.969 | 0.8 (0.3, 1.8) | 0.580 | 0.984 | 1.2 (0.5, 2.9) | 0.674 | 0.991 |
|  | MPA-binding glycan | 23 | 1.2 (0.5, 2.9) | 0.642 | 0.969 | 1.0 (0.4, 2.4) | 0.997 | 0.997 | 1.1 (0.5, 2.5) | 0.840 | 0.991 |
|  | NPA-binding glycan | 23 | 1.2 (0.5, 2.9) | 0.662 | 0.969 | 0.7 (0.3, 1.7) | 0.459 | 0.984 | 0.8 (0.4, 2.0) | 0.700 | 0.991 |
|  | PHA(E)-binding glycan | 23 | 0.6 (0.3, 1.6) | 0.327 | 0.969 | 1.0 (0.4, 2.3) | 0.942 | 0.984 | 1.0 (0.4, 2.3) | 0.987 | 0.998 |
|  | S Total group | 23 | 0.6 (0.3, 1.5) | 0.309 | 0.969 | 0.6 (0.2, 1.4) | 0.213 | 0.984 | 0.8 (0.3, 1.9) | 0.619 | 0.991 |
|  | S0 group | 23 | 1.7 (0.7, 3.9) | 0.246 | 0.969 | 1.8 (0.7, 4.2) | 0.206 | 0.984 | 1.2 (0.5, 2.8) | 0.646 | 0.991 |
|  | WGA-binding glycan | 23 | 1.5 (0.6, 3.4) | 0.376 | 0.969 | 1.5 (0.6, 3.6) | 0.359 | 0.984 | 2.0 (0.8, 4.8) | 0.132 | 0.991 |

**Supplementary Table 10.** A subset of pre-ATI lipid/metabolite biomarkers remained associated with HIV control during ATI using Spearman’s analysis. Data used for the analysis include the pooled vesatolimod and placebo arms and are limited to the analytes that pass quality control. ATI, analytic treatment interruption; FDR, false discovery rate.

| **Biomarkers** | **n** | **Spearman’s Correlation** | | | | | | | | | | | | |
| --- | --- | --- | --- | --- | --- | --- | --- | --- | --- | --- | --- | --- | --- | --- |
|  |  | **Time to 1st Rebound to  Plasma HIV-1 RNA of 200 c/mL** | | | **Time to 1st Rebound to  Plasma HIV-1 RNA of 1000 c/mL** | | | **Duration of Plasma HIV-1 RNA  ≤400 c/mL During ATI** | | | **Change in Intact Proviral HIV-1 DNA From Baseline** | | | |
|  |  | **p-Value** | **R** | **FDR** | **p-Value** | **R** | **FDR** | **p-Value** | **R** | **FDR** | **p-Value** | **R** | **FDR** |  |
| Phosphatidylethanolamine (19:1_18:1) | 22 | 0.026 | -0.47 | 0.999 | 0.045 | -0.43 | 0.998 | 0.021 | -0.49 | 0.887 | 0.332 | -0.22 | 0.937 |  |
| Phosphatidylcholine (16:0_16:0) | 22 | 0.034 | 0.45 | 0.999 | 0.013 | 0.52 | 0.998 | 0.369 | 0.20 | 0.978 | 0.083 | 0.39 | 0.937 |  |
| N6-acetyl-L-lysine | 22 | 0.028 | -0.47 | 0.705 | 0.029 | -0.46 | 0.836 | 0.198 | -0.29 | 0.954 | 0.635 | -0.11 | 0.994 |  |
| Ceramide (t17:1_24:0) | 22 | 0.095 | 0.36 | 0.999 | 0.081 | 0.38 | 0.998 | 0.015 | 0.52 | 0.887 | 0.013 | 0.53 | 0.937 |  |
| Phosphatidylcholine (20:4_20:4) | 22 | 0.090 | 0.37 | 0.999 | 0.157 | 0.31 | 0.998 | 0.022 | 0.49 | 0.887 | 0.256 | 0.26 | 0.937 |  |
| Phosphatidylinositol (19:0_18:2) | 22 | 0.189 | 0.29 | 0.999 | 0.388 | 0.19 | 0.998 | 0.028 | 0.47 | 0.887 | 0.101 | 0.37 | 0.937 |  |
| Phosphatidylcholine (16:2e_12:0) | 22 | 0.017 | 0.50 | 0.999 | 0.069 | 0.39 | 0.998 | 0.442 | 0.17 | 0.978 | 0.158 | 0.32 | 0.937 |  |
| Cholesterol ester (20:2) | 22 | 0.115 | 0.35 | 0.999 | 0.051 | 0.42 | 0.998 | 0.042 | 0.44 | 0.887 | 0.141 | 0.33 | 0.937 |  |
| Glycoursodeoxycholic acid | 22 | 0.236 | -0.26 | 0.967 | 0.063 | -0.40 | 0.964 | 0.148 | -0.32 | 0.954 | 0.762 | -0.07 | 0.994 |  |
| Phosphatidylcholine (18:3_18:3) | 22 | 0.338 | -0.21 | 0.999 | 0.607 | -0.12 | 0.998 | 0.436 | -0.17 | 0.978 | 0.394 | 0.20 | 0.938 |  |
| Phosphatidylinositol (18:0_18:0) | 22 | 0.159 | 0.31 | 0.999 | 0.395 | 0.19 | 0.998 | 0.254 | 0.25 | 0.962 | 0.312 | 0.23 | 0.937 |  |
| Phosphatidylinositol (18:0e_20:4) | 22 | 0.111 | 0.35 | 0.999 | 0.346 | 0.21 | 0.998 | 0.081 | 0.38 | 0.887 | 0.377 | 0.20 | 0.937 |  |
| Sphingomyelin (d28:0) | 22 | 0.615 | -0.11 | 0.999 | 0.879 | -0.03 | 0.998 | 0.508 | 0.15 | 0.978 | 0.944 | 0.02 | 0.992 |  |
| Trihexosylceramides | 22 | 0.269 | 0.25 | 0.868 | 0.132 | 0.33 | 0.762 | 0.082 | 0.38 | 0.629 | 0.609 | 0.12 | 0.808 |  |

**Supplementary Table 11.** A subset of pre-ATI lipid/metabolite biomarkers remained associated with HIV control during ATI using Cox proportional hazards model. Data used for the analysis include the vesatolimod and placebo groups and are limited to the analytes that pass quality control. ATI, analytic treatment interruption; CI, confidence interval; FDR, false discovery rate; HR, hazard ratio.

| **Biomarkers** | **n** | **Cox Proportional Hazards Model** | | | | | | | | |
| --- | --- | --- | --- | --- | --- | --- | --- | --- | --- | --- |
|  |  | **Time to 1st Rebound to  Plasma HIV-1 RNA of 200 c/mL** | | | **Time to 1st Rebound to  Plasma HIV-1 RNA of 1000 c/mL** | | | **Duration of Plasma HIV-1 RNA  ≤400 c/mL During ATI** | | |
|  |  | **HR (95% CI)** | **p-Value** | **FDR** | **HR (95% CI)** | **p-Value** | **FDR** | **HR (95% CI)** | **p-Value** | **FDR** |
| Trihexosylceramides | 22 | 0.32 (0.12, 0.84) | 0.021 | 0.491 | 0.30 (0.11, 0.82) | 0.018 | 0.423 | 0.28 (0.10, 0.80) | 0.017 | 0.132 |
| Phosphatidylcholine (16:0_16:0) | 22 | 0.35 (0.13, 0.92) | 0.033 | 0.499 | 0.31 (0.11, 0.83) | 0.020 | 0.677 | 0.68 (0.29, 1.62) | 0.389 | 0.735 |
| Phosphatidylcholine (20:4_20:4) | 22 | 0.51 (0.21, 1.25) | 0.140 | 0.525 | 0.64 (0.27, 1.53) | 0.319 | 0.829 | 0.25 (0.08, 0.75) | 0.014 | 0.344 |
| Phosphatidylinositol (19:0_18:2) | 22 | 0.53 (0.22, 1.29) | 0.163 | 0.544 | 0.71 (0.30, 1.69) | 0.443 | 0.851 | 0.22 (0.07, 0.67) | 0.008 | 0.344 |
| Ceramide (t17:1_24:0) | 22 | 0.79 (0.33, 1.86) | 0.585 | 0.795 | 0.75 (0.32, 1.77) | 0.510 | 0.872 | 0.38 (0.14, 0.99) | 0.048 | 0.482 |
| Phosphatidylcholine (16:2e_12:0) | 22 | 0.30 (0.11, 0.83) | 0.021 | 0.499 | 0.64 (0.27, 1.54) | 0.321 | 0.829 | 1.08 (0.45, 2.60) | 0.864 | 0.960 |
| Cholesterol ester (20:2) | 22 | 0.56 (0.23, 1.38) | 0.207 | 0.570 | 0.42 (0.16, 1.10) | 0.078 | 0.714 | 0.30 (0.11, 0.81) | 0.017 | 0.344 |
| Glycoursodeoxycholic acid | 22 | 2.28 (0.91, 5.69) | 0.079 | 0.589 | 1.74 (0.73, 4.18) | 0.213 | 0.851 | 1.66 (0.68, 4.05) | 0.263 | 0.650 |
| Phosphatidylcholine (18:3_18:3) | 22 | 1.27 (0.54, 3.01) | 0.582 | 0.792 | 1.24 (0.52, 2.93) | 0.624 | 0.906 | 1.96 (0.78, 4.95) | 0.153 | 0.534 |
| Phosphatidylethanolamine (19:1_18:1) | 22 | 1.74 (0.71, 4.27) | 0.225 | 0.581 | 2.22 (0.85, 5.80) | 0.106 | 0.735 | 2.26 (0.93, 5.51) | 0.073 | 0.482 |
| Phosphatidylinositol (18:0_18:0) | 22 | 0.88 (0.37, 2.08) | 0.771 | 0.896 | 0.94 (0.40, 2.23) | 0.887 | 0.976 | 0.71 (0.30, 1.71) | 0.445 | 0.788 |
| N6-acetyl-L-lysine | 22 | 1.63 (0.68, 3.90) | 0.270 | 0.651 | 1.25 (0.52, 2.99) | 0.620 | 0.924 | 1.33 (0.55, 3.24) | 0.525 | 0.796 |
| Phosphatidylinositol (18:0e_20:4) | 22 | 0.43 (0.16, 1.13) | 0.088 | 0.499 | 0.90 (0.38, 2.12) | 0.802 | 0.947 | 0.46 (0.19, 1.14) | 0.095 | 0.482 |
| Sphingomyelin (d28:0) | 22 | 1.08 (0.45, 2.59) | 0.861 | 0.947 | 0.86 (0.35, 2.09) | 0.741 | 0.930 | 0.88 (0.37, 2.09) | 0.774 | 0.951 |

**Supplementary Table 12.** Levels of most immune biomarkers were similar between vesatolimod and placebo at baseline and pre-ATI assessments (7/13-days post last dose). Pre-ATI was 7 days after dose 10 for plasma cytokines, 13 days after immune-cell phenotyping data. Nominal p-values are presented. ATI, analytic treatment interruption; FC, log_2_ fold change; GMFI, geometric mean fluorescent intensity; IFN, interferon; VES, vesatolimod.

| **Immune biomarkers** | **VES vs Placebo**  **Wilcoxon Rank Sum Test** | | | | **Pre-ATI vs Baseline**  **Wilcoxon Signed-Rank Test** | | |
| --- | --- | --- | --- | --- | --- | --- | --- |
|  | **Baseline** | | **Pre-ATI** | |  |  |  |
|  | **FC** | **p-Value** | **FC** | **p-Value** | **Placebo** | **VES** | **All participants** |
| CD14^+^CD16^+^ monocytes | 1.68 | 0.008 | 1.55 | 0.016 | 0.605 | 0.536 | 0.405 |
| CD16^+^ NK cells | 0.01 | 0.945 | -0.08 | 0.336 | 0.852 | 0.135 | 0.261 |
| CD38^+^CD8^+^ T cells | -0.47 | 0.181 | -0.03 | 0.804 | 0.573 | 0.211 | 0.701 |
| CD69^+^CD4^+^ T cells | -0.64 | 0.445 | 0.49 | 0.860 | 0.518 | 0.311 | 0.607 |
| CD69^+^CD8^+^ T cells | -0.07 | 1.000 | -0.10 | 0.750 | 0.755 | 0.938 | 0.701 |
| CD69^+^ NK cells | 0.18 | 0.945 | 0.13 | 0.860 | 0.852 | 0.351 | 0.309 |
| CD4^+^HLA-DR^+^CD38^+^ T cells | 0.16 | 0.945 | 0.13 | 0.547 | 0.755 | 1.000 | 0.889 |
| CD8^+^HLA-DR^+^CD38^+^ T cells | 0.07 | 0.628 | 0.33 | 0.336 | 0.852 | 0.699 | 0.944 |
| IFN-α | -1.30 | 0.259 | 0.70 | 0.741 | 0.488 | 1.000 | 0.579 |
| IL-1RA | 1.66 | 0.082 | 1.30 | 0.393 | 0.955 | 0.406 | 0.640 |
| IP10 | -0.15 | 0.804 | 0.56 | 0.076 | 0.130 | 0.201 | 0.871 |
| ITAC | -1.38 | 0.238 | -1.41 | 0.728 | 0.574 | 0.786 | 0.944 |
| CD4^+^Ki67^+^ T cells | 0.48 | 0.051 | 0.38 | 0.104 | 1.000 | 0.485 | 0.309 |
| CD8^+^Ki67^+^ T cells | 0.32 | 0.234 | 0.31 | 0.336 | 0.414 | 0.241 | 0.096 |
| pDC (CD40 GMFI) | -0.47 | 0.945 | -0.33 | 0.860 | 0.755 | 0.438 | 0.600 |
| pDC (CD54 GMFI) | 0.02 | 0.668 | 0.09 | 0.426 | 1.000 | 0.322 | 0.446 |

**Supplementary Table 13.** No significant changes were observed in HIV rebound-associated glycomic biomarkers following vesatolimod treatment. Nominal p-values are presented. ATI, analytic treatment interruption; FC, log_2_ fold change; FUC_A, antenna-fucosylated; GSL, *Griffania simplicifolia* lectin; HB, high-branched; HPA, *Helix pomatia* agglutinin; IgG, immunoglobulin G; LB, low-branched; MPA, *Maclura pomifera* agglutinin; NPA, *Narcissus pseudonarcissus* agglutinin; PHA, *Phaseolus vulgaris* phytohaemagglutinin; VES, vesatolimod.

| **Category** | **Immune biomarkers** | **VES vs Placebo**  **Wilcoxon Rank Sum Test** | | | | **Pre-ATI vs Baseline**  **Wilcoxon Signed-Rank Test** | | |
| --- | --- | --- | --- | --- | --- | --- | --- | --- |
|  |  | **Baseline** | | **Pre-ATI** | |  | **p-Value** |  |
|  |  | **p-Value** | **FC** | **p-Value** | **FC** | **Placebo** | **VES** | **All Participants** |
| **IgG**  **N-glycans** | A2B | 0.096 | 1.86 | 0.147 | 1.04 | 0.504 | 0.949 | 0.714 |
|  | A1FB | 0.169 | -0.25 | 0.186 | -0.20 | 0.247 | 0.539 | 0.346 |
|  | A2FB | 0.837 | -0.02 | 0.925 | 0.04 | 0.451 | 0.468 | 0.334 |
|  | G2FB | 0.698 | -0.04 | 0.872 | -0.01 | 0.524 | 0.350 | 0.312 |
|  | G2 | 0.750 | -0.08 | 0.628 | 0.23 | 0.350 | 0.901 | 0.802 |
| **Plasma**  **total**  **glycans** | A3F1G3S3 | 0.417 | -0.66 | 0.076 | -1.07 | 0.536 | 0.619 | 0.829 |
|  | A3G3S3 | 0.631 | 0.39 | 0.296 | 2.27 | 0.922 | 0.682 | 0.857 |
|  | FA2BG0 | 0.805 | 0.08 | 1.000 | -0.07 | 0.536 | 0.534 | 0.334 |
|  | FA2G0 | 0.581 | -0.12 | 0.605 | -0.25 | 0.955 | 0.838 | 0.910 |
|  | FA2G1 | 0.447 | -0.23 | 0.149 | -0.31 | 0.867 | 0.967 | 0.919 |
|  | FUC_A group | 0.417 | -0.71 | 0.076 | -1.06 | 0.613 | 0.820 | 0.691 |
|  | G_Total group | 0.630 | 0.01 | 0.591 | 0.02 | 1.000 | 0.885 | 0.856 |
|  | G0 group | 0.630 | -0.08 | 0.591 | -0.21 | 1.000 | 0.885 | 0.856 |
|  | G1 group | 0.298 | -0.27 | 0.115 | -0.34 | 0.694 | 0.967 | 0.733 |
|  | G2 group | 0.123 | 0.06 | 0.034 | 0.12 | 0.867 | 0.713 | 0.613 |
|  | G3 group | 0.332 | -0.28 | 0.106 | -0.43 | 0.463 | 0.838 | 0.489 |
|  | GNA-binding glycan | 0.466 | -0.06 | 0.357 | -0.05 | 0.878 | 0.967 | 0.886 |
|  | GSL_II | 0.438 | 0.11 | 0.561 | -0.14 | 0.721 | 0.221 | 0.448 |
|  | HB group | 0.581 | -0.19 | 0.115 | -0.36 | 0.336 | 1.000 | 0.581 |
|  | HPA-binding glycan | 0.466 | 0.30 | 0.333 | -0.28 | 0.574 | 0.361 | 0.801 |
|  | LB group Plasma | 0.581 | 0.02 | 0.115 | 0.05 | 0.297 | 1.000 | 0.570 |
|  | MPA-binding glycan | 0.238 | 0.11 | 0.628 | -0.09 | 0.505 | 0.340 | 0.613 |
|  | NPA-binding glycan | 0.265 | -0.02 | 0.401 | 0 | 0.574 | 0.744 | 0.965 |
|  | PHA(E)-binding glycan | 0.265 | -0.02 | 0.149 | 0.03 | 0.065 | 0.917 | 0.223 |
|  | S_Total group | 0.680 | 0.04 | 0.213 | 0.07 | 0.536 | 0.775 | 0.694 |
|  | S0_group | 0.680 | -0.15 | 0.213 | -0.24 | 0.536 | 0.775 | 0.694 |
|  | WGA-binding glycan | 0.825 | 0.01 | 0.747 | 0 | 0.878 | 0.59 | 0.818 |

**Supplementary Table 14.** No significant changes were observed in HIV rebound-associated lipid/metabolite biomarkers following vesatolimod treatment. Nominal p-values are presented. ATI, analytic treatment interruption; FC, log_2_ fold change; VES, vesatolimod.

| **Immune biomarkers** | **VES vs Placebo**  **Wilcoxon Rank Sum Test** | | | | **Pre-ATI vs Baseline**  **Wilcoxon Signed-Rank Test** | | |
| --- | --- | --- | --- | --- | --- | --- | --- |
|  | **Baseline** | | **Pre-ATI** | |  |  |  |
|  | **FC** | **p-Value** | **FC** | **p-Value** | **Placebo** | **VES** | **All Participants** |
| Glycoursodeoxycholic acid | -0.03 | 0.783 | -0.43 | 0.368 | 0.156 | 0.421 | 0.113 |
| Phosphatidylcholine (16:0_16:0) | -0.04 | 1.000 | 0.01 | 0.783 | 0.469 | 0.890 | 0.588 |
| Phosphatidylcholine (18:3_18:3) | -0.04 | 0.945 | -0.67 | 0.032 | 0.078 | 0.561 | 0.524 |
| Phosphatidylcholine (20:4_20:4) | -0.15 | 0.447 | -0.25 | 0.123 | 0.938 | 0.303 | 0.371 |
| Phosphatidylethanolamine (19:1_18:1) | -0.12 | 0.581 | -0.17 | 0.630 | 0.688 | 0.489 | 0.949 |
| Phosphatidylinositol (18:0_18:0) | -0.09 | 0.407 | 0.51 | 0.162 | 0.938 | 0.135 | 0.262 |
| Phosphatidylinositol (19:0_18:2) | -0.01 | 1.000 | -0.22 | 0.267 | 0.813 | 0.151 | 0.235 |
| N6-acetyl-L-lysine | -0.07 | 0.680 | -0.06 | 0.680 | 0.375 | 0.083 | 0.046 |
| Ceramide (t17:1_24:0) | 0.16 | 0.891 | 0.06 | 0.945 | 0.813 | 0.524 | 0.799 |
| Phosphatidylcholine (16:2e_12:0) | -0.07 | 0.630 | -0.14 | 0.581 | 0.688 | 0.135 | 0.176 |
| Phosphatidylinositol (18:0e_20:4) | 0.07 | 0.783 | -0.10 | 0.680 | 0.937 | 0.454 | 0.633 |
| Cholesterol ester (20:2) | -0.06 | 0.680 | -0.04 | 0.731 | 0.469 | 0.561 | 0.321 |
| Sphingomyelin (d28:0) | -0.32 | 0.837 | 0.37 | 0.891 | 0.937 | 0.169 | 0.248 |
| Trihexosylceramides | -0.25 | 0.162 | -0.22 | 0.298 | 0.813 | 0.679 | 0.824 |

**Supplementary Figure 1.** Protein synthesis–related pathways were associated with HIV rebound. Dot size represents the number of hits. Dot color represents upregulation (red, mean fold-change >1), downregulation (blue, mean fold-change <1), and nonsignificant (gray). Fold change is shown on the *x*-axis. Nominal p-values were used in the analysis. BL, baseline; cp, copies; tRNA, transfer RNA.


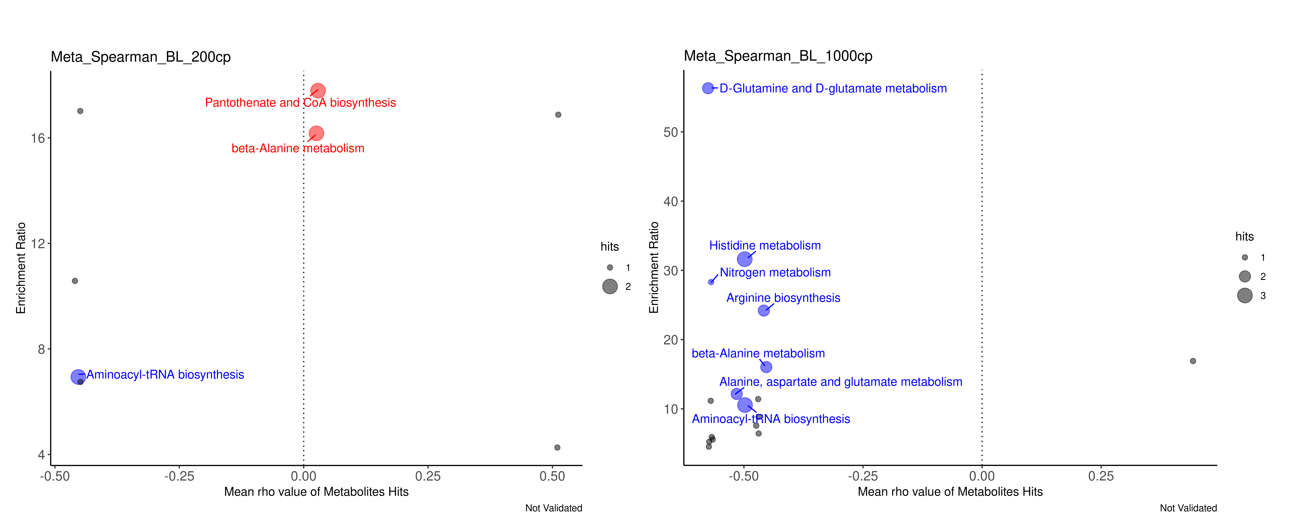


**Supplementary Figure 2.** Pro-inflammatory tryptophan metabolism and protein synthesis–related aminoacyl-tRNA biosynthesis were enriched after vesatolimod treatment. Online pathway enrichment analysis was referred to a library of 84 metabolite sets based on KEGG human metabolic pathways (Oct. 2019). [https://www.metaboanalyst.ca](https://www.metaboanalyst.ca/) Enrichment ratio is computed by observed hits/expected hits; nominal p-values are presented. ATI, analytic treatment interruption; KEGG, Kyoto Encyclopedia of Genes and Genomes; tRNA, transfer RNA; VES, vesatolimod.

*Asterisk denotes significant change.


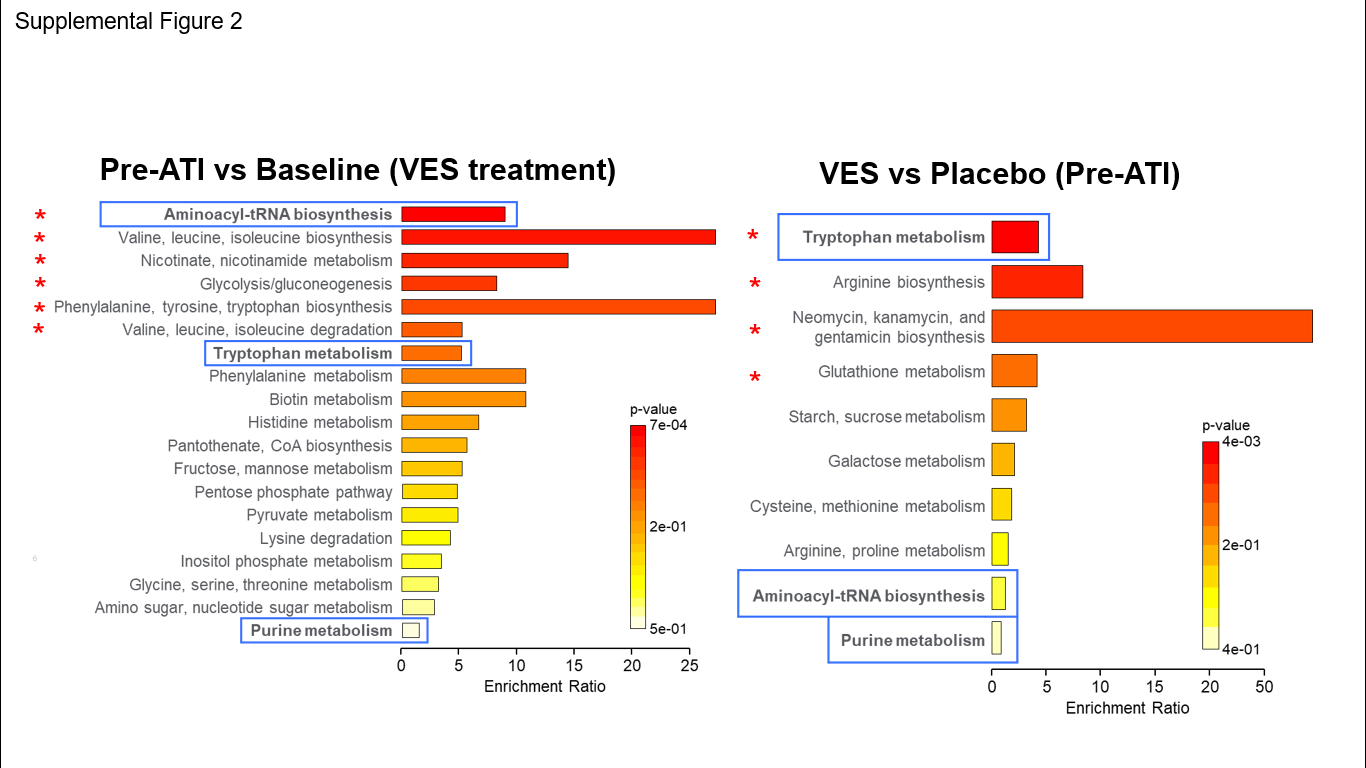


**Supplementary Figure 3.** Vesatolimod mediated significant changes in more lipid subclasses than placebo. Significant lipids were identified with Wilcoxon signed-rank test between baseline and pre-ATI. ATI, analytic treatment interruption; PLB, placebo; VES, vesatolimod.


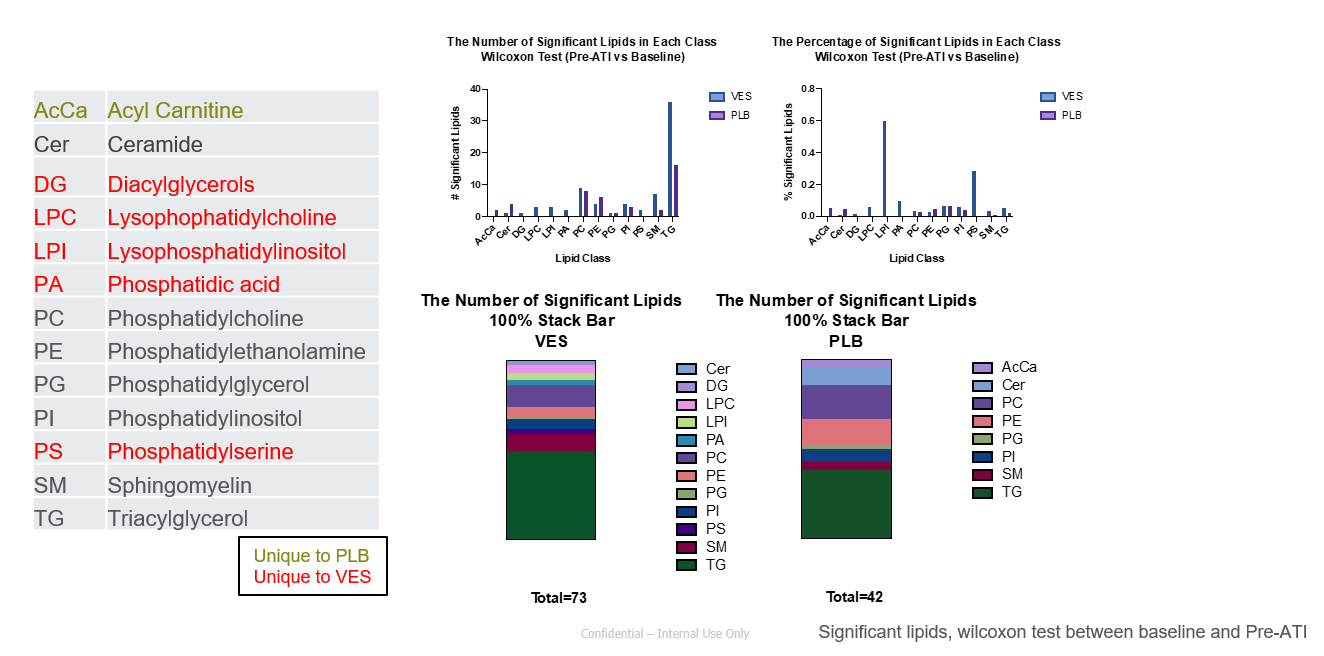

Supplement: Supplementary file 1 [file DataSheet_1.docx]
